# Static Disorder has Dynamic Impact on Energy Transport in Biomimetic Light-Harvesting Complexes

**DOI:** 10.1021/acs.jpcb.2c06614

**Published:** 2022-10-03

**Authors:** Leo M. Hamerlynck, Amanda J. Bischoff, Julia R. Rogers, Trevor D. Roberts, Jing Dai, Phillip L. Geissler, Matthew B. Francis, Naomi S. Ginsberg

**Affiliations:** † Department of Chemistry, University of California Berkeley, Berkeley, California94720, United States; ‡ Department of Physics, University of California Berkeley, Berkeley, California94720, United States; § Kavli Energy NanoSciences Institute, Berkeley, California94720, United States; ∥ Chemical Sciences Division, Lawrence Berkeley National Laboratory, Berkeley, California94720, United States; ⊥ Materials Sciences Division, Lawrence Berkeley National Laboratory, Berkeley, California94720, United States; # Molecular Biophysics and Integrated Bioimaging Division, Lawrence Berkeley National Laboratory, Berkeley, California94720, United States

## Abstract

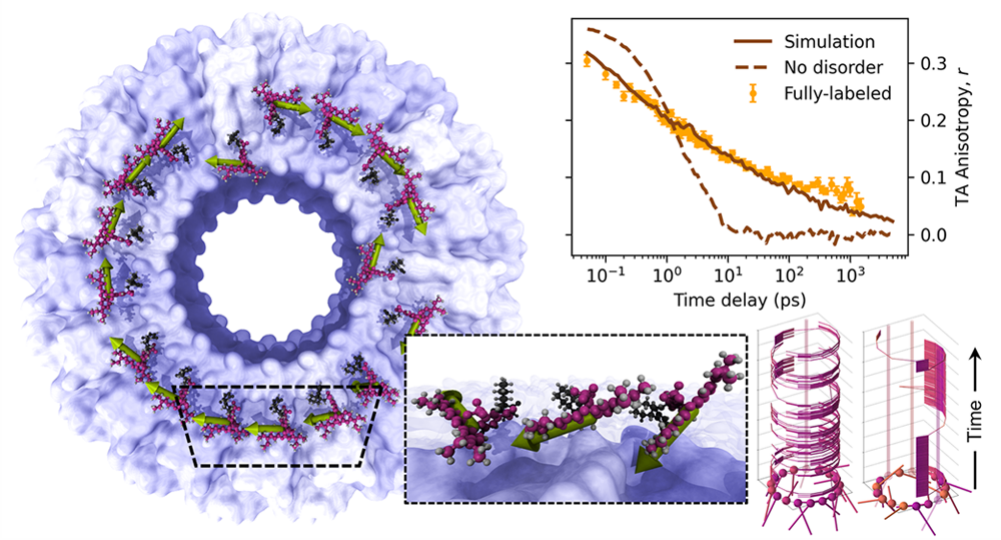

Despite extensive studies, many questions remain about what structural and energetic factors give rise to the remarkable energy transport efficiency of photosynthetic light-harvesting protein complexes, owing largely to the inability to synthetically control such factors in these natural systems. Herein, we demonstrate energy transfer within a biomimetic light-harvesting complex consisting of identical chromophores attached in a circular array to a protein scaffold derived from the tobacco mosaic virus coat protein. We confirm the capability of energy transport by observing ultrafast depolarization in transient absorption anisotropy measurements and a redshift in time-resolved emission spectra in these complexes. Modeling the system with kinetic Monte Carlo simulations recapitulates the observed anisotropy decays, suggesting an inter-site hopping rate as high as 1.6 ps^–1^. With these simulations, we identify static disorder in orientation, site energy, and degree of coupling as key remaining factors to control to achieve long-range energy transfer in these systems. We thereby establish this system as a highly promising, bottom-up model for studying long-range energy transfer in light-harvesting protein complexes.

## Introduction

Natural light-harvesting protein complexes (LHC) in plants and photosynthetic bacteria absorb photons from the sun and, within a few tens of picoseconds, transport that energy across several protein complexes to reaction centers where charge separation and subsequent chemistry occur.^1^ Remarkably, this transfer process can have near unity quantum efficiency. Thus a detailed model of which parameters lead to this efficiency is of great fundamental interest and is of value for developing efficient artificial light-harvesting technologies such as sensitive photodetectors and artificial photosynthesis.^2−4^ Despite extensive studies, a full picture of the structure-function relationships that give rise to the excellent energy transfer capabilities of LHCs in plants and photosynthetic bacteria remains to be described. In natural LHCs, the difficulty lies mainly in the complexity of these multi-process systems, compounded by their fragility *in vitro*, which makes systematic investigation, for example, through targeted mutation,^5−8^ difficult.

Many artificial LHCs have been developed and studied, but these typically rely on short-range energy funnels.^9−13^ Light harvesting in natural LHCs often involves energy transfer between identical chromophores. In particular, some purple bacteria species develop extended regions of identical protein subunits in low-light conditions, which is known to increase efficiency.^2,14^ Measurement of energy transfer between spectrally identical chromophores is difficult due to the lack of an obvious signature of energy transfer. In some cases, time-resolved polarization anisotropy measurements can be used to characterize energy transfer,^15−18^ by measuring the loss of preferred orientation, that is, depolarization, of the probed transition dipole moment (TDM) following excitation. Transient absorption anisotropy (TAA) is one technique able to probe depolarization without relying on fluorescence. These types of measurements are challenging due to the low sensitivity of absorption measurements and often overlapping spectral features with uniquely evolving anisotropies, explaining the relative dearth of reports in the literature compared to other forms of ultrafast spectroscopy.^19,20^ The benefit of using TAA, however, is its general applicability to any absorbing sample and the relative simplicity of ultrafast implementation compared to, for example, fluorescence upconversion. In addition, TAA as a quantitative measure of energy transfer relies on the ability to isolate the depolarization associated with energy transfer from those due to other processes, such as rotational diffusion. Through such study, we demonstrate that the cpTMV complex is capable of energy transfer, with a very high inter-chromophore transfer rate, and characterize its full extent. In natural systems, the inability to modularly synthesize control complexes, however, still makes this isolation difficult.

**Figure 1:**
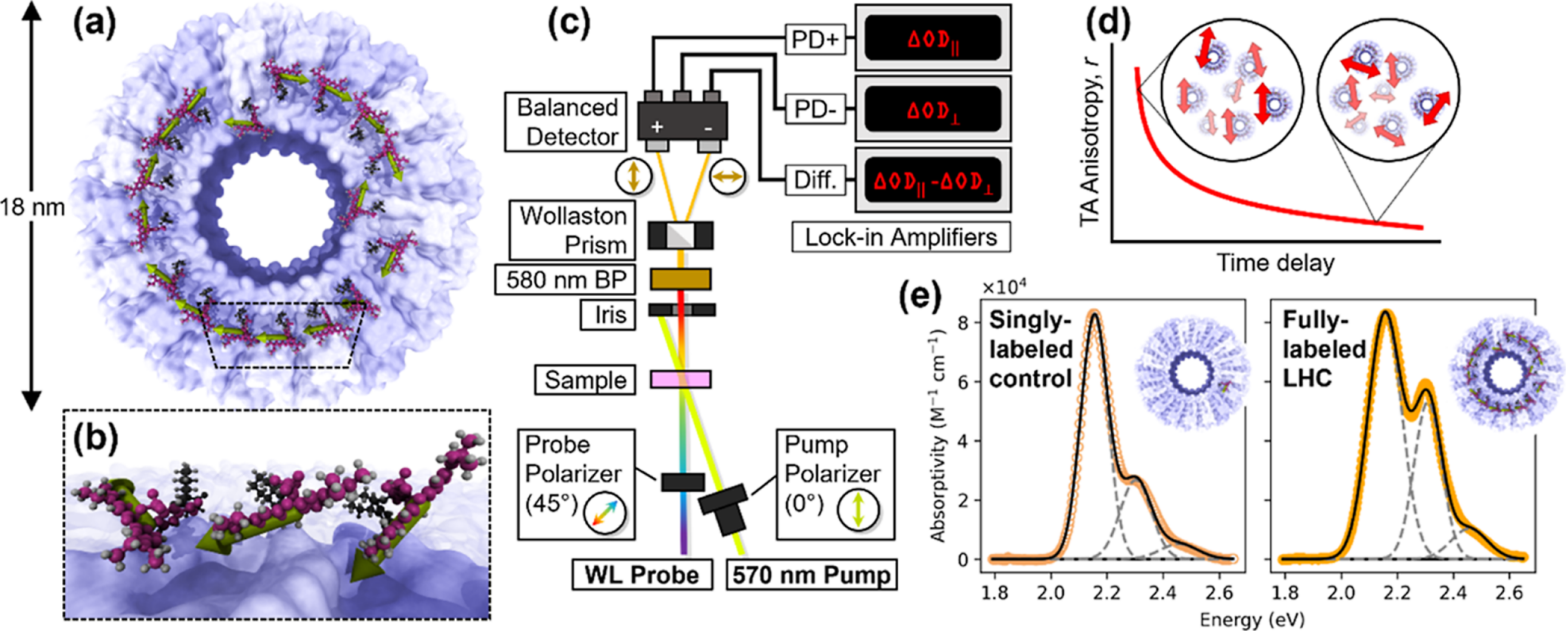
Overview of the experiment. (a) Schematic representation of the cpTMV biomimetic LHC with attached SRB chromophores and (b) a zoomed-in view on three binding sites demonstrating the high TDM orientational disorder. (c) Diagram of the TAA experimental setup, indicating polarization of pump and probe beams and use of Wollaston prism to measure both polarization components simultaneously, yielding (d) TAA decay curve following optical excitation as energy transfer randomizes TDM orientation. (e) Steady-state absorption spectra of the singly labeled controls and the fully labeled LHCs.

In this study, we demonstrate rapid energy transfer in a modular biomimetic LHC consisting of protein-bound identical chromophores and uncover the microscopic sources of disorder whose future control will facilitate even longer-range exciton migration in this system. This system is based on a circular permutant of the tobacco mosaic virus (cpTMV) coat protein, which self-assembles into C_2_-symmetric double-disk super-complexes (PDB:3KML),^21^ with each disk composed of 17 identical monomers, as seen in Figure 1a. By preparing mutants with reactive cysteine substitutions, we covalently attach chromophores at specific sites, as illustrated in Figure 1b, affording great control over factors such as chromophore identity and location^22^ and the rigidity of the linking molecule.^23^ Utilizing this modularity, we construct a model system amenable to study by TAA, and importantly, we can also generate singly labeled control complexes to isolate the signature of energy transfer in this measurement from confounding effects of rotational diffusion. This work represents a crucial step in constructing an artificial LHC super-complex that can serve as a truly biomimetic model system to study long-range energy transfer. Our measurements demonstrate that site-to-site energy transfer occurs quickly in these LHCs, while long-ranged transport is currently somewhat impeded by disorder in TDM orientation, site energy, and coupling. Fortunately, the modularity afforded by this system affords a high degree of control appropriate for addressing these sources of disorder.

## Materials and Methods

### Preparation of cpTMV Artificial Light-Harvesting Complexes

The synthesis of the biomimetic cpTMV LHCs, including synthetic procedures, mutant generation, protein expression, bioconjugation, and purity analyses, has been described in detail previously.^23^ Detailed information on mutant generation, characterization, and modification of the previously unreported mutants generated for this work, cpTMV-D18C, cpTMV-V32C, and cpTMV-S65C, can be found in the Supporting Information. Briefly, maleimide-functionalized sulforhodamine B chromophores with SS-cyclohexyl linkers are synthesized from commercially available alcoholamines. Site-directed mutagenesis is performed on a circular permutant of the tobacco mosaic virus coat protein to prepare D18C mutants, and the proteins are expressed in *E. coli*. cpTMV fully labeled LHCs and singly labeled control samples are prepared by maleimide bioconjugation of the modified SRB complexes with the reactive cysteine introduced to the mutant cpTMV surface. Fully labeled LHCs are prepared by performing the bioconjugation with >1 equiv of modified SRB and the mutant cpTMV monomers. For the singly labeled controls, 0.01 equiv is used, resulting in statistically singly labeled complexes, though the majority have no attached chromophores. Labeling yields are verified by electrospray ionization time-of-flight mass spectrometry connected with high-performance liquid chromatography (ESI-TOF LC-MS) (see Figures S1 and S2), and size-exclusion chromatography is performed to purify the samples. Prior to TAA measurements, all samples are diluted to an optical density (OD) of 0.1 at the pump wavelength.

By varying the stoichiometry of the bioconjugation sample preparation procedure, we produce both fully labeled LHCs (Figure 1e, right), with a chromophore at every site, and singly labeled control complexes (Figure 1e, left), which allows us to study inter-chromophore interactions separate from protein-chromophore interactions. Their corresponding steady-state absorption spectra in Figure 1e both show a vibronic progression of peaks. The higher relative amplitude of the second vibronic peak in the fully labeled complex most likely arises from the close proximity of the SRB molecules to one another.^24^ We rely on a separation of the timescales of energy transfer and chromophore reorientation, using short, rigid linking molecules and a sufficiently immobile attachment site on the protein, in order to distinguish energy transfer from rotational diffusion of the chromophore. For the purpose of immobilization, we select D18C TMV mutants in which the chromophore binding sites are 4.5 nm from the center of the disk and separated by 1.5 nm from one another. D18C mutants also demonstrate the greatest immobilization in control experiments, as seen in the comparison of TAA decays of singly labeled controls at different sites in Figure S3. With an inter-site distance of 1.5 nm, the inter-chromophore energy transfer is in the regime of Förster resonance energy transfer (FRET).^25−27^ Besides proximity, efficient FRET requires good spectral overlap between the donor fluorescence and acceptor absorption, as well as favorable TDM orientation. In the absence of energy transfer, TAA measurements on the SL control complexes report solely on the motion of the chromophore and how it is impacted by interaction with the protein. While this does not account for the effects that chromophore–chromophore interactions may have on reorientation in the fully labeled LHCs, it is unlikely that the increased concentration of chromophores would lead to significantly faster reorientation dynamics.

### Ultrafast Transient Absorption Anisotropy

We measure the ultrafast optical TAA of our biomimetic LHCs to characterize the ultrafast depolarization associated with energy transfer following optical excitation by a linearly polarized 100 fs laser pulse. TAA is a variation of transient absorption (TA), a pump-probe technique, in which a pump light pulse (Figure 1c, yellow) first excites the sample (Figure 1c, pink), and a probe light pulse (Figure 1c, rainbow-colored) is used to observe changes of the excited state population at progressively longer time delays in successive experiments.^28^ Experimentally, the anisotropy *r*(*t*) obtained from a TAA measurement is given by  where ΔOD_∥_ and ΔOD_⊥_ are the transient changes in optical absorption with the probe polarization oriented parallel and perpendicular to the pump, respectively,^28^ as denoted with double-headed arrows in Figure 1c. Energy transfer between two differently oriented chromophores changes the orientation of the probed TDM, leading to orientational decorrelation and thus a measurable depolarization of the transient absorption signal, as demonstrated schematically in Figure 1d as the red decaying curve and corresponding scrambling of the red TDM double-headed arrows at later time delay. In the simplest case, TAA is related to the orientation of the probed TDMs of the population of chromophores by , where θ is the angle between the probed TDM and the excitation polarization, and *r*_0_ is the initial anisotropy, which, due to photoselection with linearly polarized light, cannot exceed 0.4.^29^

**Figure 2:**
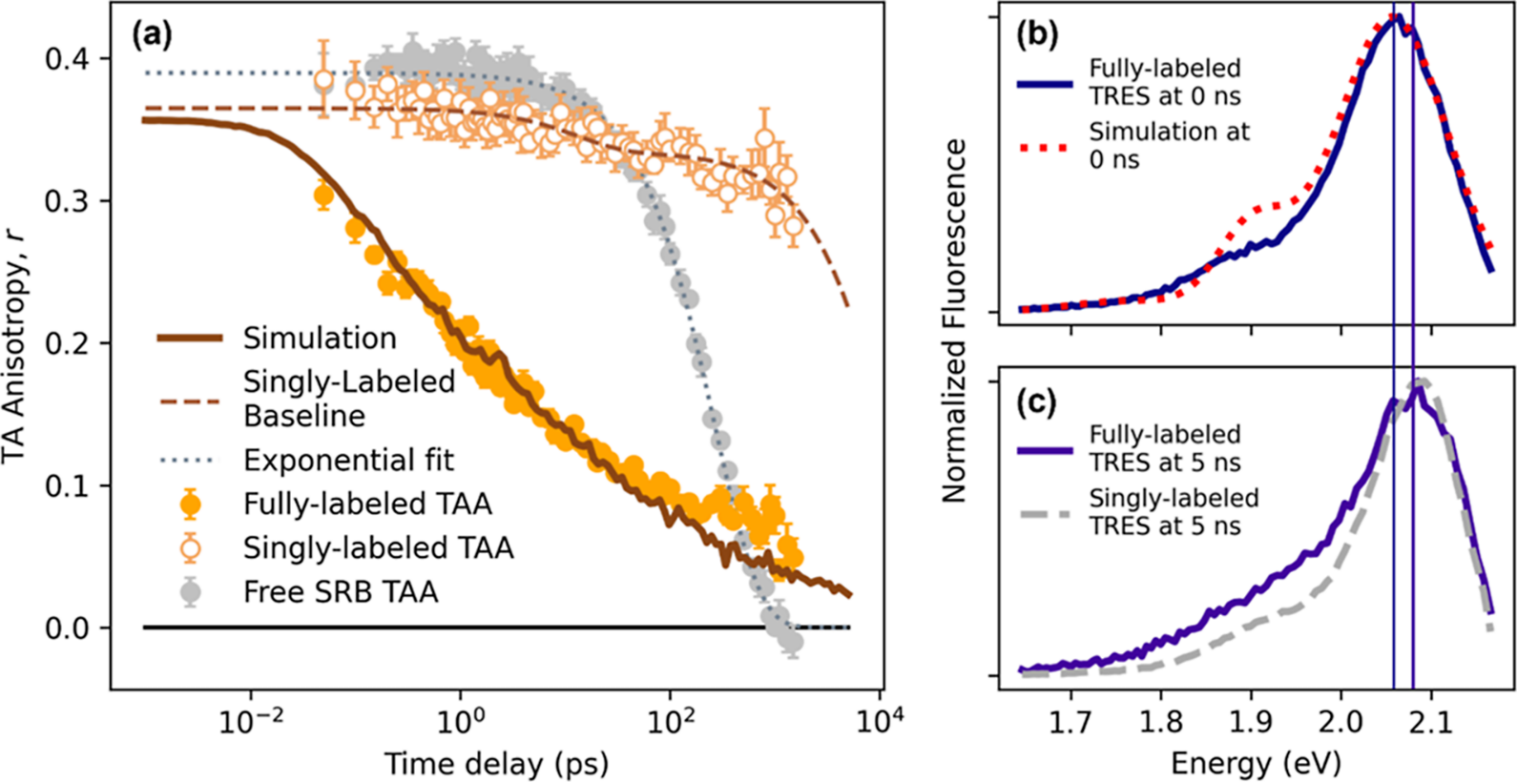
Spectroscopically resolving energy transfer in the LHC. (a) Experimental TAA data (dots) at 580 nm of free SRB chromophore in solution (gray), singly labeled control (orange outlined) and fully labeled LHC (solid orange), with error bars representing standard error relative to the mean of 10 consecutive measurements. Note the log scale in time, causing exponential decays to appear as sigmoids. Overlaid are the biexponential fit to the singly labeled decay (dashed line), anisotropy computed from the simulation (solid curve), and an exponential fit to the free SRB decay (dotted curve). (b) Early-time emission spectrum of fully labeled LHC, overlaid with predicted spectrum from simulation (dotted red), and (c) late-time emission spectrum of fully labeled LHC overlaid with late-time emission spectrum of singly labeled controls (dashed gray), with drop lines to indicate spectral shift.

We perform ultrafast TAA measurements using a Ti:sapphire Coherent Legend regenerative amplifier that outputs 800 nm, 80 fs pulses at a repetition rate of 5 kHz, and a total power of 5 W. The fundamental beam is split, and in one path, we generate 570 nm, 100 fs pump pulses in a home-built noncollinear optical parametric amplifier, followed by a dual prism compressor and a chopper set to 1/8 the fundamental frequency, that is, 625 Hz. On the other path, we generate the white-light probe by focusing the 800 nm fundamental into a CaF_2_ crystal that is continuously rastered back and forth to avoid burning. The polarization of the pump and probe are set at 45° relative to one another with a Glan–Thompson prism polarizer and a linear film polarizer, respectively, placed immediately before the sample to avoid any depolarizing effects of reflection. We note that thermal drift in the polarizing optics can cause slight misalignment, which may cause slight error in the measurement. One example of this is the free SRB TAA decay shown in Figure 2a, which appears to fall slightly below 0. This feature does not appear to impact the analysis. See Supporting Information for additional details. Following the sample, a series of irises removes as much scattered light as possible, and a 580 ± 5 nm bandpass filter selects the measurement wavelength on the red side of the ground state bleach (GSB) peak of sulforhodamine B (SRB), the labeling chromophore. The GSB, which results from the absence of ground-state absorption in the excited chromophores, is the strongest feature in the TA spectrum of the SRB molecule. In addition, because the pumped and probed transitions are the same in the GSB, this transition is expected to have the maximum initial anisotropy of 0.4.^28^ The sample is rastered back and forth during the experiment to prevent sample damage, and steady-state UV/vis measurements are taken before and after each measurement to ensure no sample degradation has occurred. Following the bandpass filter, a Wollaston prism rotated 45° relative to the probe polarization splits the probe beam into its two polarization components, which are incident on the two photodiodes of a Thorlabs PDB210 balanced photodetector (BPD). The BPD outputs three voltage signals *P*_+_, *P*_–_, and *D*, respectively, proportional to the intensities of the parallel and perpendicular probe components and their difference. We measure these values by lock-in amplification referenced to the frequency of the chopper, yielding differential (pump-on minus pump-off) values Δ*P*_+_, Δ*P*_–_, and Δ*D*, which provides excellent signal-to-noise ratio. The anisotropy is then calculated with the expression , where *G* is the gain factor  accounting for an additional amplification in the difference signal. A derivation of this expression is provided in the Supporting Information.

### Time-Resolved Emission Spectroscopy

To characterize the evolution of the average site energy following excitation, we also collect time-resolved emission spectra (TRES) of the fully labeled LHCs and singly labeled controls. We make these measurements on a PicoQuant FluoTime FT-300 fluorometer. The samples are resonantly excited at 565 nm by a 100 ps laser pulse from a PicoQuant D-TA-560B diode source, and an emission decay is collected via time-correlated single photon counting at wavelengths from 570 to 750 nm, producing a set of time-resolved spectra with hundreds of picoseconds resolution.

### Kinetic Monte Carlo Simulations

**Figure 3:**
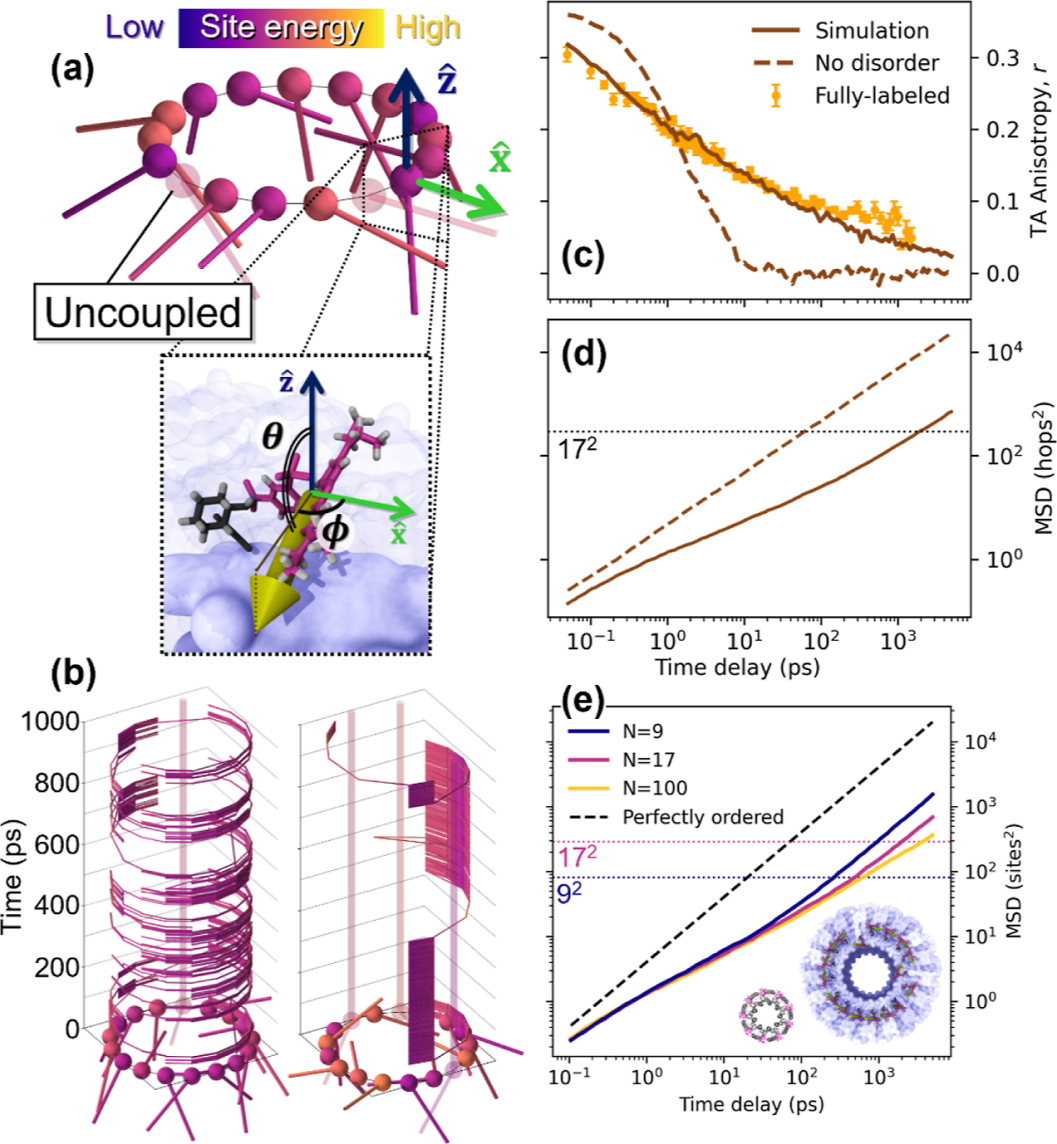
Overview of simulation setup and results. (a) Representative ring of TDM orientation vectors, with site energy color scale normalized to entire population and uncoupled chromophores shown with reduced opacity; zoom-in shows a schematic representation of the chromophore corresponding to a given TDM vector in the site-local coordinate frame. (b) Two such rings with excitation trajectories shown, with time increasing vertically upward. (c) The simulation fit with and without disorder included, overlaid with experimental TAA data, and (d) corresponding mean squared expansion of excitation population, with a dotted line indicating the boundary of the equilibrium regime. (e) Similar such MSD curves (solid) shown for simulations with varying numbers of sites, including *N* = 9 corresponding to LH2 (shown inset, PDB:1NKZ),^44^ demonstrating size-dependent final diffusivity when compared to simulations without disorder (dashed), the latter of which appear identically as shown for all values of *N*.

To relate the observed TAA and TRES data to a microscopic picture of energy transfer within the TMV complexes, we use a kinetic Monte Carlo simulation based on FRET-like incoherent hopping of excitations among static TDMs on a ring. The kinetic Monte Carlo simulations are described in detail in Figure S4, and the accompanying text is given in the Supporting Information. Our approach incorporates methods developed by Bradforth *et al.*(15) and Bardeen.^30,31^ We represent an artificial LHC as a ring of unit vectors, each representing the orientation of the TDM of a chromophore bound to the protein surface. A representation of one such ring is shown in Figure 3a, with the TDMs represented by rods originating at the chromophore binding sites. The TDM orientation local coordinate system is shown in the inset. The vectors are represented in a lab frame with the *z*-axis parallel to the implied polarization of the excitation. The TDM orientations are sampled according to the probability distribution obtained from molecular dynamics simulations of a single chromophore attached to three surface α-helices of the cpTMV complex reported previously.^23^ We use the lateral orientation of the xanthene core as a proxy for the orientation of the TDM.^32^ Each site is assigned an energy drawn from a Gaussian distribution with a width σ_ih_, and intrinsic site absorption and emission spectra are constructed using Gaussian fits of the experimental steady-state absorption and emission spectra. The rate of energy transfer between each site is then calculated using Förster theory. A subset is randomly selected as uncoupled chromophores by reducing the rates of transfer to and from these sites by 0.001. 10,000 such rings, shown in Figure S5 to be sufficient to reach convergence, are generated and randomly rotated to simulate the random orientation of cpTMV complexes in solution, and an excitation is placed on one site on each chromophore, with site selection weighted by its TDM’s alignment with the *z*-axis aligned electric field. The excitations are then allowed to hop around each ring according to the relative probabilities of pairwise computed hopping probabilities, using an implementation of the Gillespie algorithm.^33−35^ From these trajectories, we compute the anisotropy, mean squared displacement (MSD), and the ensemble spectrum as a function of time after excitation.

## Results and Discussion

As shown in Figure 2a on a logarithmic time scale, TAA measurements of the GSB of the cpTMV LHCs (solid yellow circles) demonstrate decay, while uncoupled singly labeled control complexes (open yellow circles) exhibit almost none. The TA anisotropy decay of the cpTMV LHCs extends over several decades in time, from hundreds of femtoseconds to hundreds of picoseconds, with super-exponential decay at early times and then stretched, sub-exponential decay at late times. In the singly labeled control complexes, where energy transfer is not possible, the TA anisotropy remains almost constant over the course of the measurement, falling only slightly from 0.37 immediately after excitation to 0.33 at 1.5 ns. In a free dye control sample (Figure 2a, grey circles), we observe an exponential decay from 0.38 to 0 (a characteristic sigmoid shape when plotted on a logarithmic time axis) with a time constant of 260 ps, which matches well to literature values.^37,38^ We generate the magic-angle TA decays ΔOD_MA_ = ΔOD_∥_+2·ΔOD_⊥_ for the denominator of the anisotropy expression. As shown in Figure S6, the magic-angle TA decays for the free dye and singly labeled complexes are well-fit by a biexponential decay, which we previously attributed to vibrational relaxation and radiative decay processes.^23^ Meanwhile, the LHCs show a more extended TA decay profile over several decades that decays faster than their singly labeled counterparts. Table S1 lists the parameters for multiexponential fits of these data.

The TRES data demonstrate the emergence of a red-shifted component in the fully labeled LHCs that is absent in the singly labeled controls. The fully labeled LHC TRES data are fit by three exponentially decaying components, which are shown in Figure S7: one short-lived ∼310 ps component that is redshifted by 0.04 eV relative to the singly labeled control spectrum, one long-lived component resembling that of the SL controls (2.85 ns), and one intermediate component (1.27 ns) that appears to be a mixture of the two with an intermediate lifetime. Thus, the spectrum appears red-shifted relative to the singly labeled controls at early times, as seen in Figure 2b, while at late times the spectrum, as shown in Figure 2c, appears very similar to that of the singly labeled control, which does not demonstrate any appreciable shift in energy over time. We assign the red-shifted components to the excitations that undergo rapid energy transfer and the SL-like component to excitations on uncoupled chromophores that are unable to participate in energy transfer.

For the simulation, to account for contributions to the anisotropy decay in the model that are not due to inter-chromophore energy transfer, we incorporate the effects observed in the singly labeled control complexes. Doing so involved fitting the experimental singly labeled control TAA data, as described in the following process. We fit the slight TAA decay of the singly labeled control complexes with a biexponential decay, following the model of segmental motion of a protein-bound chromophore.^36^ The expression used is  where τ_F_ and τ_P_ are the timescales of the fast chromophore motion and slow protein motion, respectively, *r*_0_ is the initial anisotropy, and 0 ≤ α ≤ 1 parameterized the chromophore’s freedom of motion relative to the protein, with 0 being fully immobilized and 1 being fully unconstrained. Through least-squares fitting, we arrive at parameters of *r*_0_ = 0.36, τ_F_ = 130 ps, τ_P_ = 14 ns, and α = 0.09, producing the dashed red “baseline” fit shown in Figure 2a. The combination of the two exponential decay components results in an apparent plateau in the singly labeled TAA decay. The initial anisotropy value *r*_0_ of the singly labeled complexes is lower than the theoretical maximum of 0.4; this is true not only in the SL control complexes but also in the free chromophore controls. This lowered anisotropy is likely because of a small (<10%) contribution from the stimulated emission signal at this wavelength, which is not guaranteed to have maximum intrinsic anisotropy as the GSB is. In addition, scattering of the pump or the probe beam can spuriously increase or decrease the measured anisotropy. While we took steps to mitigate this, we note that the initial anisotropy can vary between ∼0.36 and 0.39 between different measurements but without an appreciable impact on the rest of the analysis. See the Methods section for further discussion.

We take the anisotropy decay of the SL controls to represent the contribution to the decay in the fully labeled LHCs coming from chromophore and protein fluctuations, occurring independent of any energy transfer. When two processes act independently to rotate the observed TDM, the effects of the two processes on the anisotropy decay combine multiplicatively. Thus, the anisotropy decay from the simulation *r*_sim_(*t*) and the decay from the SL control data *r*_SL_(*t*) are combined to yield a fit to the fully labeled TAA decay . The factor of 0.4 is necessary to avoid doubly applying the effect of photoselection, which is present in both *r*_sim_(*t*) and *r*_SL_(*t*). This final expression combining the raw simulation results and the singly labeled control fit is shown in Figure 2a as the solid red curve, demonstrating excellent agreement with the experimental data.

The key simulation parameters required to recapitulate the experimental anisotropy decay are those that affect the sites differently on each ring, that is, those that introduce disorder: the static TDM orientation distribution, the site energy, and the uncoupled chromophores. Together, these three sources of disorder cause the anisotropy decay to be stretched from the exponential decay expected in the absence of disorder, as seen in Figure 3b. Each type of disorder has a distinct effect on the anisotropy and spectral properties of the population, but we observe a common signature when we track the MSD of the excitation in hops^2^ over time. Specifically, we compute the MSD by tracking the number of sites hopped clockwise (the negative direction) and counterclockwise (the positive direction), effectively unraveling the excitation trajectories around the rings. The introduction of each type of disorder generates two different regimes in the MSD versus time. Diffusive transport is always arrived at at later times; the apparent subdiffusive behavior in the exciton migration that precedes it corresponds, however, to a non-equilibrium regime in which the initial condition relaxes to a more stable state through migration-based exploration of the chromophore disorder. This effect manifests as a distinct “s-curve” shape in the solid red plot of MSD versus time in Figure 3d. In the non-equilibrium regime, the mean squared expansion slows over time, seen as the slope of the curve temporarily decreasing over the first few hundred ps. In the diffusive transport regime, the curve adopts a linear form with a unity slope, also benchmarked with the dashed line in Figure 3d, as the diffusivity *D* on this periodic one-dimensional lattice is governed by the relationship MSD(*t*) = 2*Dt*. Although each type of disorder qualitatively manifests similarly in the average observed MSD versus time, we next review their individual contributions to the anisotropy and transport dynamics and explore their impact as a function of the extent of each type of disorder.

**Figure 4:**
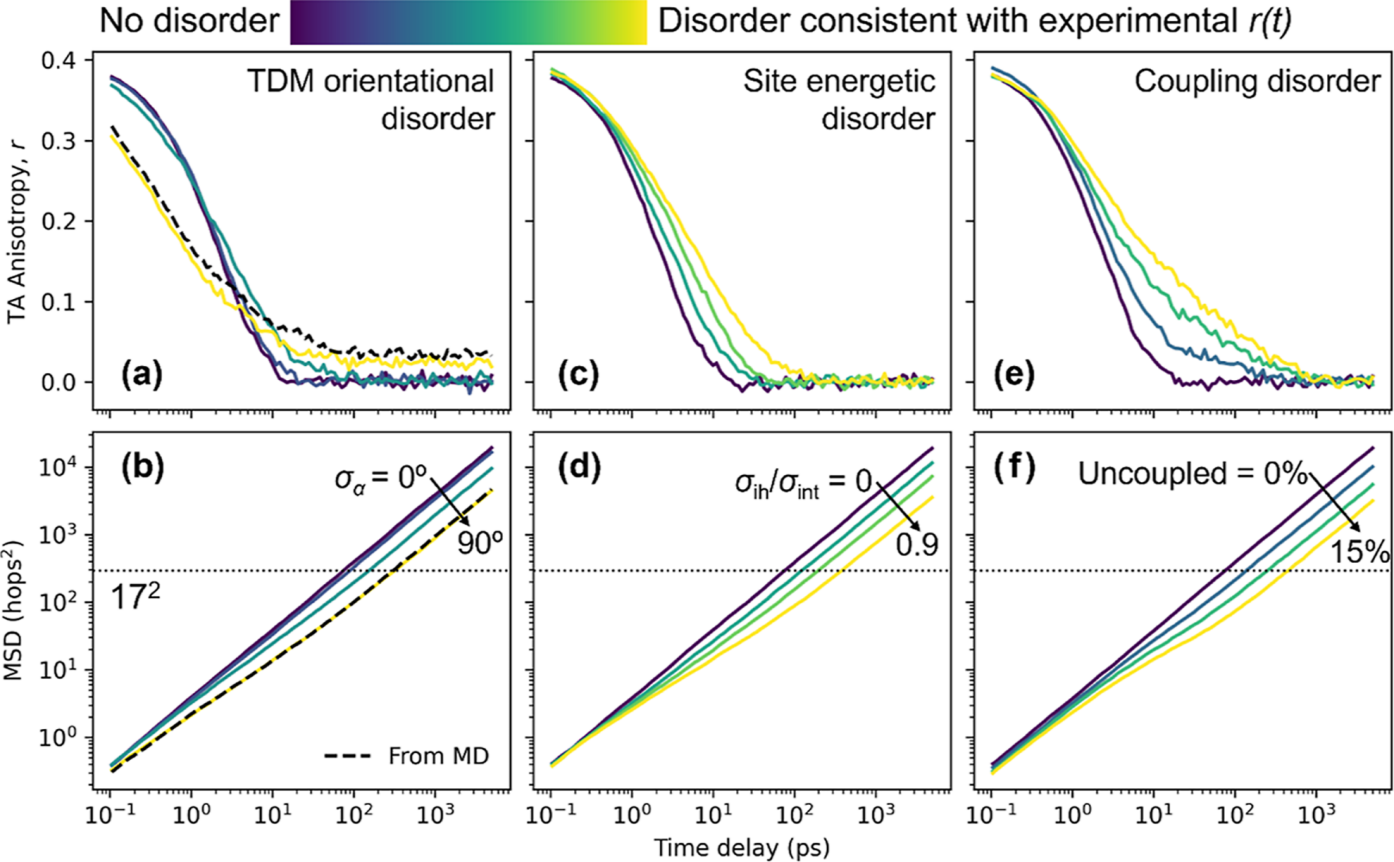
Simulated energy transfer observables in the LHC. Simulated TAA (a,c,e) and MSD (b,d,f) on populations including disorder in only one parameter: (a,b) TDM orientation, using a Gaussian orientation distribution with width σ_α_, (c,d) site energy, and (e,f) coupling. Normalized color scale is from complete absence of disorder increasing to equivalent value as included in reported simulation fit. TDM orientational plots (a,b) also include the results using molecular dynamics distribution (black dashed curves).

TDM orientational disorder causes the anisotropy to decay more slowly at late time delays as more relative TDM orientations are sampled. To explore this further, we additionally run simulations in which each TDM orientation is sampled from a Gaussian probability distribution centered on the orientation  with width σ_α_, as shown in Figure 4a,b. Except in cases of particularly poor dipole–dipole coupling, such as when adjacent TDMs are orthogonal, increased TDM orientational disorder reduces exciton mobility over time. In Figure 4b, we see that from the case with no disorder (σ_α_ = 0°) to the highly disordered limit (σ_α_ = 90°), the equilibrium excitation diffusivity decreases from 1.9 hops^2^/ps (blue curve) to 0.44 hops^2^/ps (yellow curve). On the log–log scale of Figure 4b, the difference in diffusivity manifests as an offset between two linear curves with unity slope in the diffusive regime, that is, at time delays when the curves have exceeded the indicated MSD=(17 hops)^2^ dotted line. A unique effect of TDM orientational disorder, however, is an increased anisotropy decay at early times, as seen in the σ_α_ = 90° case (yellow curve) in Figure 4a, where the anisotropy is lesser than in the perfectly ordered case (dark blue) until approximately 4 ps. This effect is not related to a change in the exciton mobility but is due to the greater depolarization associated with each hop between sites. To most faithfully reconstruct the experimental data, we use a TDM orientation probability distribution constructed from molecular dynamics simulations of an SRB-like dye interacting with key protein α-helices of the LHC construct^23^ (see Supporting Information). This simulation-derived distribution generates the anisotropy and MSD versus time delay shown in the black dashed curves in Figure 4a,b, respectively. We find, however, that any sufficiently broad distribution produces similar kinetics, as the results using the σ_α_ = 90° Gaussian distribution (yellow curve) match almost identically the results using the molecular dynamics simulations. A slight offset in the anisotropy of these curves in Figure 4a evidences a very slight difference in the intrinsic anisotropy of these populations. Thus, the orientation distribution used is not a unique solution to fit the experimental data, but rather a limit in which the orientations are distributed broadly, and our model is thereby constrained to this limit by the prediction of molecular simulation.

Site energetic disorder similarly slows the exciton mobility over time, as excitons accumulate in the lowest-energy sites, often separated spatially from one another, similar to effects reported previously in other site-energetically disordered systems.^39,40^ From the simulation, we also predict a spectral red shift as the excitations “cool” while exploring the energetic landscape. In the simulation, we parameterized the site energy disorder as the ratio of the (inhomogeneous) width of the ensemble’s energy distribution to the intrinsic width of each individual site, σ_ih_/σ_int_, where the values are constrained by the width of the measured absorption peak. A modest amount of site energy disorder, σ_ih_/σ_int_ = 0.9, produces a redshift of 0.04 eV that agrees with that seen in the TRES data, as shown by the overlap of the simulated spectrum (dotted red curve) and the early-time fluorescence of the biomimetic LHC (solid blue curve) in Figure 2b. In Figure 4d, the equilibrium exciton diffusivity decreases from 1.9 hops^2^/ps with no site energetic disorder (blue curve) to 0.35 hops^2^/ps (yellow curve/faint dotted line) when σ_ih_/σ_int_ is increased to 0.9—the ratio ultimately deduced to best match the experimental data. This effect is primarily evidenced in the later decades of the anisotropy decay in Figure 4c, causing a progressively longer tail to appear as site energy disorder is increased relative to the exponential decay expected in the absence of disorder. If, however, the only contributions to disorder were orientational and this modest contribution from site energies, the model could not mimic the long tail of the experimental anisotropy decay. In fact, to fit these data by increasing the site energy disorder alone would require σ_ih_/σ_int_ = 4. In order to simultaneously model the TAA decay and the TRES data, another source of disorder is required—hence the addition of randomly uncoupled chromophores.

The introduction of uncoupled chromophores slows the overall exciton mobility considerably and greatly stretches the late-time anisotropy decay. Uncoupled chromophores, that is, sites to and from which the rate of energy transfer is greatly diminished, are introduced to the simulation phenomenologically to account for the singly labeled-like fluorescence and long-time anisotropy observed in the experimental data. With the abovementioned orientational disorder constrained to the highly random limit and site energy disorder constrained by the magnitude of the redshift observed in TRES, we find that uncoupling 15% of the chromophores results in an excellent fit to the TAA data. As seen in Figure 4f, when increasing this coupling disorder from 0 to the 15% used in the best fit to the experimental data, the equilibrium excitation diffusivity decreases from 1.9 hops^2^/ps to 0.31 hops^2^/ps. The exciton mobility is reduced due to the barrier-like nature of these uncoupled chromophores, which effectively introduce gaps in the ring of sites. In addition, the subpopulation of excitations that are initialized on one of these isolated chromophores remains locked in said state, meaning this subpopulation remains highly polarized for much longer, which greatly slows the late time anisotropy decay. This is best demonstrated by comparing the coupling and site energy disorder. Despite a similar impact on exciton mobility, that is, the vertical offset in the diffusion regime for the highest amounts of disorder (yellow curves) in Figure 4d,f, the effect of coupling disorder seen in Figure 4e on the anisotropy is highly substantial, with an elevated tail stretching into the 100s of picoseconds, compared to the modest impact of site energetic disorder on anisotropy in Figure 4c.

## Discussion

Natural LHCs achieve exquisite control over factors such as chromophore energy and orientation, and yet disorder remains present in natural LHCs despite their capacity for long-range energy transfer.^41^ The impact of this disorder on long-range energy transfer, whether positive or negative,^10,39−43^ is critical to elucidate. Overall, we find from our model that static disorder has the effect of “stretching” out the relaxation of the biomimetic LHC system (and the associated anisotropy decay) across several decades in time from an exponential form, in the case of no disorder, to that which is observed in the TAA data. Our simulations allow us to study this effect in detail *in silico*. They show that in the presence of all types of disorder, the electronically excited state reaches thermal equilibrium after a period of a few hundred picoseconds, as evidenced by the MSD adopting a linear dependence on time, albeit with a lower diffusion constant than the perfectly ordered case. As demonstrated in Figure 3d, the time at which this equilibrium diffusion is first observed corresponds to the time at which, on average, all excitations have explored their entire ring, that is, MSD=(17 hops)^2^ (dotted horizontal line). Thus, this behavior is associated with an equilibration as excitons explore the entirety of the system and its associated disorder, a phenomenon also observed in a linear system by Ahn *et al.*(30) This slowing of exciton mobility following excitation manifests in the anisotropy as an elongation of the model’s decay (solid curve) in later decades (Figure 3c) as average exciton migration in the equilibrium state is strictly slower than the initial condition. Effectively, long-range migration is dominated by the slowest transfer steps, whether resulting from a pair of poorly aligned TDMs, a site that is low in energy relative to its neighbors, or an uncoupled chromophore. This effect is well illustrated in the rightmost simulation trajectory in Figure 3b, where despite fast inter-site hopping, the trajectory shows the excitation remaining in limited subsections of the ring for long periods of time, only rarely hopping between these subsections, such as near 100, 500, and 900 ps. Closer inspection reveals the barriers between wells in this case to be both high-energy sites (colored yellow) and uncoupled chromophores (translucent spires). In contrast, the leftmost example in Figure 3b shows a trajectory on a ring in which site energy disorder is low (all site energies are close to the mean energy, indicated by a common plum color of the trajectory trace) and there is only one uncoupled chromophore. Indeed, on this ring, the excitation travels more freely, with only two noticeable exceptions: one clearly due to the uncoupled chromophore, and also a second, more subtle orientational barrier due to the TDM seven sites counterclockwise to the first, which is nearly orthogonal to both its neighbors, corresponding to κ^2^ values near 0. Simulations of rings of chromophores with fewer sites, with all else being equal, illustrate that this effect is dependent on the number of sites in a ring. A system with fewer sites recovers diffusive behavior earlier and at a higher final equilibrium diffusivity, as seen by comparing the late-time diffusivities between a 9-membered ring (blue) and a 17-membered ring (magenta) in the MSD versus time plots in Figure 3e. Because of the finite number of sites on the rings in question, the excitations on any given ring each only sample that particular ring’s realization of disorder, which cannot represent the entirety of the disorder in the continuous distribution from which it is generated. In the simulation, once all sites on all ring realizations of a given size have been fully explored by the excitation population, the population cannot relax any further and thus adopts a higher diffusivity than would be observed on an infinite 1D lattice with equivalent disorder. This effect is more pronounced for fewer-membered rings simply due to the more limited sampling of disorder for any given realization. In comparison, in the absence of disorder, the diffusivity does not change between rings of different sizes, as we would expect. Based on this observation, we hypothesize that incoherent intra-complex energy transfer in circular light-harvesting complexes with fewer sites is more robust to disorder in general, compared to larger complexes. This principle could help to explain the design of the bacterial antenna complexes, as LH2 displays only nine-fold rotational symmetry,^44^ and in low-light or low-temperature conditions, some purple bacteria instead produce LH4, an eightfold-symmetric structure. Excitonic coupling and coherent transport do occur in these complexes, but nevertheless, this principle could apply to any steps involving incoherent transport. Including inter-complex energy transfer would diminish this effect, as it opens the collection of accessible sites beyond the closed periodic system of our model. Nevertheless, when complex-to-complex transfer is rate-limiting, efficient diffusion around a closed ring would be important for encountering exit sites.

The most straightforward demonstration of the slowing mobility occurs due to static site energy disorder, as the site energy is not directly correlated with the TDM orientation distribution as compared to the other two sources of disorder. A disordered energy environment intuitively results in the emergence of energy “wells,” that is, low-energy sites, out of which energy transfer is slower because it is more likely to be uphill. The population begins with the same average spectral overlap as a system with no site energetic disorder and thus similar exciton mobility. As the excitons explore their chromophore rings, however, they tend to become trapped in these low-energy sites, adopting an equilibrium Boltzmann distribution and decreasing the spectral overlap and, correspondingly, the mobility, until equilibrium is reached. Thus, increasing site energetic disorder does not affect the early-time anisotropy decay, but does stretch the late-time decay. Site energetic disorder can additionally be revealed spectrally from the red-shifted component in the TRES measurements. Since the magnitude of the TRES redshift is determined by the extent of energy disorder, we constrain the latter to a value of 0.04 eV, corresponding to σ_ih_/σ_int_ = 0.9, to recapitulate the experimental redshift. A limitation of our model, however, is the assumption that site energy is independent of chromophore orientation. Since interaction with the protein surface can certainly impact electronic configuration, this is an approximation. In the case of strong interaction between chromophore and protein,^45^ this assumption would likely not hold and would require more detailed rate calculations, which would greatly increase the complexity. Yet, this zeroth-order approximation appears valid in our case, as evidenced by the very modest differences between the steady-state spectra of free chromophores and singly labeled control complexes.

We demonstrate that more care must be taken when associating TAA data with energy transfer when there is high static TDM orientational disorder in the experimental system, as the TDM orientation is itself associated with the anisotropy signal. We find that a high degree of static orientational disorder exists in the surface-bound chromophores, as predicted by molecular dynamics simulations. Despite the rigidity of the linker holding the chromophores steady on nanosecond timescales, which we have shown previously to be important for extending the chromophore’s initially hot vibrational excited state,^23^ there is a high degree of orientational disorder that is static on the time scale of the electronic dynamics. This disorder is necessary in the simulations to recapitulate the early-time anisotropy decay, which, as seen in the highly disordered case (yellow curve) of Figure 4a, occurs super-exponentially, that is, faster than the exponential decay of the perfectly ordered case (blue curve), due to the high degree of depolarization associated with a single hop In particular, in this case of high static orientational disorder, there is an increase in the rate of early-time anisotropy decay relative to a perfectly ordered system, due to increased depolarization with each site-to-site hop. Nevertheless, static orientational disorder leads to a similar decrease in exciton diffusivity over time as is observed with energetic disorder. This relationship may be less intuitive for orientation disorder than for energetic disorder, as the symmetry of the κ^2^ term in the FRET rate between two chromophores implies that it is symmetric in the forward and reverse directions, precluding “trapping” on an individual site. As a result of the orientational disorder, however, there emerge groups of consecutive sites with favorable dipole orientation, and the (relatively) low κ^2^ at the boundaries of these groups has a trapping effect on excitations that enter these “wells.” Indeed, over a population with a highly disordered Gaussian TDM orientational probability distribution of width σ_α_ = 90°, the average of the minimum κ^2^ between neighbors on each ring is 2% of the average of all such κ^2^ values. So, as excitations migrate and explore the orientational space, over time they tend to spend more time trapped in these subsections of the ring rather than freely diffusing, which limits the long-range mobility of excitations. Once the excitations have fully explored the space, however, we recover diffusive transport limited by the relatively slow well-to-well hopping rather than the faster site-to-site transfer. Again in the σ_α_ = 90° case, the final diffusivity is 23% that of the long-time diffusivity obtained for σ_α_ = 0°. In comparison, natural LHCs precisely control the orientations of their chromophores through steric effects and hydrogen bonding with specifically placed residues; we believe that this fine control over orientation is necessary for efficient long-range energy transfer. This reflects what Sarovar and Whaley have studied in detail—that control over orientation is essential to optimize energy transfer in the presence of disorder in such LHCs.^46^

The lack of configurational control of the chromophores, that is, their physical configuration relative to the protein, in this system is what we hypothesize to be the source of disorder in the degree of coupling between chromophores, which similarly impacts long-range energy transport by producing barriers to transport. The introduction of this disorder to our model is justified phenomenologically. First, it captures the singly labeled-like fluorescence component observed in the fully labeled LHCs. Second, the long-lived anisotropy cannot be accounted for by orientational and energetic disorder alone. The subpopulation of initially excited chromophores that are poorly coupled to the majority of others would fluoresce identically to the singly labeled controls and would remain highly anisotropic over the course of the TAA measurement. A lack of control over the position of the chromophore could manifest this disorder, leading some chromophores to adopt positions sufficiently far from the others to greatly reduce FRET coupling due to the 1/*R*^6^ dependence. As an illustrative example, Figure 1a shows two chromophores which are anomalously configured to reside near the pore of the protein ring, which would place them approximately 1.5x the distance from their neighbors as those residing far from the pore. Adopting a configuration that places more protein between the chromophores would further reduce the FRET coupling due to protein’s typically higher index of refraction compared to water (1.66 vs 1.33) and the 1/η^4^ dependence of the FRET rate. Empty sites on the complexes could serve a similar purpose as uncoupled chromophores by presenting a barrier to energy transfer but would not produce the observed singly labeled-like fluorescence signal at long time delays. In terms of energy transfer, the effect of these uncoupled chromophores is similar to that of orientational disorder, effectively dividing the ring into wells. Escaping such a well requires bypassing an uncoupled chromophore via next-nearest-neighbor hopping.

Thus far, our model suggests an encouraging capacity for remarkably fast site-to-site energy transfer in the synthetic LHC, but that it is limited in range due to ensemble disorder. This fast hopping rate, averaging 1.6 ps^–1^_,_ is due to the 1.5 nm site-to-site separation, which places the chromophores (with an average *R*_0_ of 6.5 nm) close enough together to allow FRET efficiencies in excess of 99%. Viewed in isolation, this site-to-site rate is in fact faster than the incoherent hopping rate of 0.67 ps^–1^ reported between B800 molecules in antenna complexes of *Rhodobacter acidophila*,^17^ demonstrating the strong potential of the synthetic LHC (though slower than the coherent energy transfer rate of >13 ps^–1^ in the B850 aggregate). As counterpoints, as can be seen in Figure 3c, these hops occur on limited subsets of the ring due to barriers presented by static disorder, and a well-known side effect of placing chromophores close together without the benefit of photosynthesis’ billions of years of pigment-protein complex evolution is nonradiative contact quenching. Indeed, we observe signatures attributable to moderate quenching in both transient absorption and fluorescence, seen as an increase in the rate of signal decay relative to the singly labeled controls. In singly labeled controls, the TA signal decays with a slow component of 1.8 ns, while in the fully labeled LHCs we observe a multiexponential decay with two slow components of 97 and 870 ps, as listed in Table S1. In natural light-harvesting systems, it is not fully understood how such quenching is avoided. In such systems, however, chromophores are most commonly found in pockets within the protein, not covalently linked to the surface. In our biomimetic LHCs, then, it appears the same lesser control over chromophore orientation and position that impacts long-range exciton migration could also play a role in allowing this aggregation and subsequent quenching to occur.

## Conclusions

Regardless, our findings suggest that protein scaffolds such as cpTMV, combined with judicious chromophore conjugation, are promising systems for both studying and imitating the efficiency of energy transfer in LHCs. In this study, we have demonstrated energy transfer in a modular biomimetic LHC with inter-chromophore energy transfer rates slower than but comparable to natural systems and used kinetic Monte Carlo simulations to describe the key sources of disorder affecting energy transfer in this system. Through the modularity of the system, these key sources of disorder that must be controlled to optimize energy transfer in biomimetic LHCs are site energy, chromophore orientation, and chromophore coupling. While we showed previously that rigid linkers are capable of slowing vibrational relaxation, which can contribute to improved energy transfer, this study demonstrates that control over the specific chromophore orientation and configuration relative to the protein and to other chromophores remains critical to optimize exciton migration. In particular, fine control over the configuration of chromophores, as occurs in natural systems, will be necessary to prevent the poorly coupled chromophores that we identified have to hinder exciton migration. Furthermore, we uncovered a potential design principle to ensure robustness to disorder in systems relying on exciton diffusion on a discrete periodic lattice, as in purple bacteria. We find that a smaller period (number of sites on a ring) allows the photoexcitation to equilibrate more rapidly in the presence of disorder, reducing the impact of disorder on long-range energy transfer.

Based on the abovementioned findings, one could evaluate and improve the energy transfer performance of model light-harvesting complexes. First, the significant role of static disorder in affecting long-range energy transport points to the need to develop biomimetic LHC strategies beyond restricting chromophore motion and focused on specifically and repeatedly orienting chromophores in the biomimetic LHC structure. For instance, placing chromophores inside a protein cavity might soon become possible by breaking the C_2_ symmetry of a disk-like structure like the one employed here. This type of advance would enable investigation of systems more closely resembling natural LHCs. Second, the extensibility of biomimetic LHCs across many scales of organization, from individual chromophore-protein systems, to fully labeled isolated LHCs, to supercomplexes of LHCs arrayed in films or rod geometries, will continue to enable the study of interactions involved in long-ranged light harvesting through a bottom-up approach. For example, coupling biomimetic LHCs together asymmetrically, allowing for the creation of supercomplexes of different types of disks (*e.g.*, “donor” and “acceptor” disks), might further mirror the membrane-bound systems found in light-harvesting bacteria. Furthermore, combining TAA and TRES with new techniques, such as transient microscopy, should allow for energy transfer in such super-complexes to be tracked directly in space, building a ground-up picture of efficient energy transfer in LHCs and heterogeneous systems more broadly and over more scales than is possible with time-resolved spectroscopy alone.

## Supporting Information Available

Detailed description of the sample preparation and characterization; more details on the TAA measurement and kinetic Monte Carlo simulations; TAA data on singly labeled controls at the site used and at other sites; and magic-angle TA kinetics of D18C cpTMV complexes (PDF)
