# Supplementary material for: Static Disorder has Dynamic Impact on Energy Transport in Biomimetic Light-Harvesting Complexes
Source: J Phys Chem B. 2022 Oct 3;126(40):7981–91. doi: 10.1021/acs.jpcb.2c06614 (PMC9574921; doi:10.1021/acs.jpcb.2c06614)
Supplement: Supplementary file 1 — jp2c06614_si_001.pdf [file jp2c06614_si_001.pdf]

Supporting Information for: Static disorder has dynamic impact on energy transport in biomimetic light-harvesting complexes

Leo M. Hamerlynck[1,6], Amanda J. Bischoff[1,6], Julia R. Rogers*[1], Trevor D. Roberts[1], Jing Dai†[1], Phillip L. Geissler[1,4], Matthew B. Francis[1,5,6], and Naomi S. Ginsberg‡[1,2,3,4,5,6]

[1]Department of Chemistry and [2]Department of Physics, University of California Berkeley, Berkeley, California 94720, United States
[3]Kavli Energy NanoSciences Institute, Berkeley, California 94720, United States
[4]Chemical Sciences Division, [5]Materials Science Division, and [6]Molecular Biophysics and Integrated Bioimaging Division, Lawrence Berkeley National Laboratory, Berkeley, California 94720, United States

1 Sample Preparation and Characterization

1.1 Steady-state Spectroscopy

UV-Vis absorption measurements were conducted on a Cary UV-Vis 100 spectrophotometer (Agilent, USA). Slit widths were set to 1.0 nm for both excitation and emission. Protein concentration was routinely determined by UV/Vis analysis on a Nanodrop 1000 instrument (Nanodrop, USA) by monitoring absorbance at 280 nm.

1.2 Mass Spectrometry (MS)

Proteins and their bioconjugates were analyzed using an Agilent 1200 series liquid chromatograph (Agilent Technologies, USA) that was connected in-line with an Agilent 6224 Time-of-Flight (TOF) LC/MS system equipped with a Turbospray ion source. Protein samples were run with a Proswift RP-4H column (Dionex, USA). Protein mass reconstruction was performed on the charge ladder with Mass Hunter software (Agilent, USA).

1.3 High Performance Liquid Chromatography (HPLC)

HPLC was performed on Agilent 1100 Series HPLC Systems (Agilent, USA). Sample analysis for all HPLC experiments was achieved with an in-line diode array detector (DAD) and in-line fluorescence detector (FLD). Size exclusion chromatography (SEC) was performed using a Polysep-GFC-P-5000 column (4.6 x 250 mm) (Phenomenex, USA) at 1.0 mL/min using a mobile phase of 10 mM sodium phosphate buffer, pH 7.2.

1.4 Construction of cpTMV expression plasmids

The gene of the circular permutant of TMV (cpTMV) was produced using standard molecular biology techniques based on a gene for the coat protein of the TMV U1 strain optimized for the codon usage of E. coli (Genscript, Piscataway, NJ) as reported by Dedeo *et al.*[1] Point mutations were introduced using

*Current address: Department of Systems Biology, Columbia University, New York, New York, 10032, United States
†Current address: Antibody Engineering, Genentech, Inc, 1 DNA Way, South San Francisco, CA 94080, USA
‡Author to whom correspondence should be addressed. E-mail: nsginsberg@berkeley.edu

QuikChange mutagenesis (Stratagene, Santa Clara, CA). The following sets of primers were used for the D18C, V32C, and S65C mutations:

D18C:
Sense: 5' GGATGCGACCCGTCGTGTGGATTGTGCCACCGTGGCGATTCG 3'
Antisense: 5' GCTGCGAATCGCCACGGTGGCACAATCCACACGACGGGTCGC 3'
V32C:
Sense: 5' CGCAGCGCCATCAATAACCTGATTTGCGAACTGATTCGTGGC 3'
Antisense: 5' CCGGTGCCACGAATCAGTTCGCAAATCAGGTTATTGATGGCGC 3'
S65C:
Sense: 5' CCCGGCGGGCGAAGGCAGCTATTGCATTACCACCC 3'
Antisense: 5' GCTCGGGGTGGTAATGCAATAGCTGCCTTCGCCCCG 3'

1.5 Protein expression and purification

The expression and purification of cpTMV proteins is based on a previously reported procedure with minimal modifications.[2] BL21-CodonPlus (DE3)-RIL competent cells (D18C) or BL21 (DE3) Tuner competent cells (V32C, S65C) were transformed with the vectors described above. Colonies were selected for inoculation in 1 L Terrific Broth with 100 µg/L ampicillin at 37 °C. When cultures reached an optical density of 0.6 to 0.8, 100 µL 0.3 M isopropyl-β-D-thiogalactopyranoside (IPTG) was added. After growing for 23 h at 30 °C (D18C) or 20 °C (V32C, S65C), the cells were harvested by centrifugation (8000 rpm, 30 min) and the cell pellet was stored at -20 °C After freezing, cell pellets were partially thawed and resuspended in 10 mL lysis buffer, 20 mM triethanolamine (TEA) pH 7.2. Cells were lysed by sonication with a 2 s on, 4 s off cycle for a total of 10 minutes using a standard disruptor horn at 60% amplitude (Branson Ultrasonics, Danbury, CT). The resulting lysate was cleared at 14,000 rpm for 30 minutes. The supernatant was treated with 30-40% volume (3-4 mL) of saturated ammonium sulfate and allowed to rotate for 10 minutes at 4°C to allow for complete protein precipitation. The precipitated protein was collected at 11,000 rpm for 30 minutes and resuspended in 10 mL lysis buffer. Complete re-dissolving of the protein and removal of residual ammonium sulfate was accomplished by performing dialysis in 1 L lysis buffer overnight with at least one buffer exchange. The resulting protein solution was treated with 5 µL benzonase (MilliporeSigma, St. Louis, MO) and 4 mg $MgCl_2$ at room temperature for 30 minutes before the solution was spun down at 10,000 rpm for 10 minutes. The resulting supernatant was filtered through a 0.22 µm filter and purified using an anionic DEAE column and purified with a 0 – 180 mM NaCl gradient elution in 20 mM TEA buffer, pH 7.2. The fractions containing cpTMV were further purified using a Sephacryl S-500 column in 10 mM NaPhos pH 7.2 elution buffer. Pure fractions were collected and concentrated using Amicon Ultra 100 kD MWCO centrifugal concentrators (MilliporeSigma, St. Louis, MO). Purity was confirmed by SDS-PAGE and ESI-TOF-MS, and assembly state was confirmed by HPLC-SEC (**Fig. S1**). Protein was flash frozen and stored indefinitely at -80°C or stored for no longer than 2 weeks at 4°C without observed decomposition or change in assembly state.

1.6 General procedure for labeling cpTMV with sulforhodamine B

Labeling of cpTMV was accomplished according to a previously published procedure with modification.[2] To 100 µL of cpTMV (100 µM in 10 mM sodium phosphate buffer, pH 7.2) was added 10 equiv. tris(2-carboxyethyl)phosphine (TCEP). The reaction mixture was briefly agitated and then incubated in 1.5 mL Eppendorf tubes for 1 h. After 1 h, the TCEP was removed with a NAP-5 Sephadex G-25 column (GE Healthcare, USA), and the flow-through was either added directly to a 1.5 mL Eppendorf tube containing 5 equiv. 2-(6-(diethylamino)-3-(diethyliminio)-3H-xanthen-9-yl)-5-(N-((1S,2S)-2-(2,5-dioxo-2,5-dihydro-1H-pyrrol-1-yl)cyclohexyl)sulfamoyl)benzenesulfonate (SRB-CycSS-mal) (for 100% labeling) or to an empty 1.5 mL Eppendorf tube followed immediately by the addition of 0.01 equiv. of SRB-CycSS-mal (for 1% labeling). The reaction mixture was briefly agitated and then incubated in 1.5 mL Eppendorf tubes at room temperature with an aluminum foil cover. After 2 h, the crude reactions were purified with two NAP-5 Sephadex G-25 columns (GE Healthcare, USA) to remove the excess chromophores. The fractions that showed absorption at 280 nm were combined and subjected to repeated (5x) spin concentration using 100 kDa MWCO filters for further removal of chromophores. The protein conjugates were analyzed with HPLC-SEC for assessment

of purity and validation of assembly state. The labeled samples were adjusted to a final concentration of 0.1 absorbance at the SRB absorption maximum (565 nm) using a Cary UV-Vis 100 spectrophotometer prior to TAA measurements. The mass spectra of SRB-labeled cpTMVs are shown in **Fig. S2**.

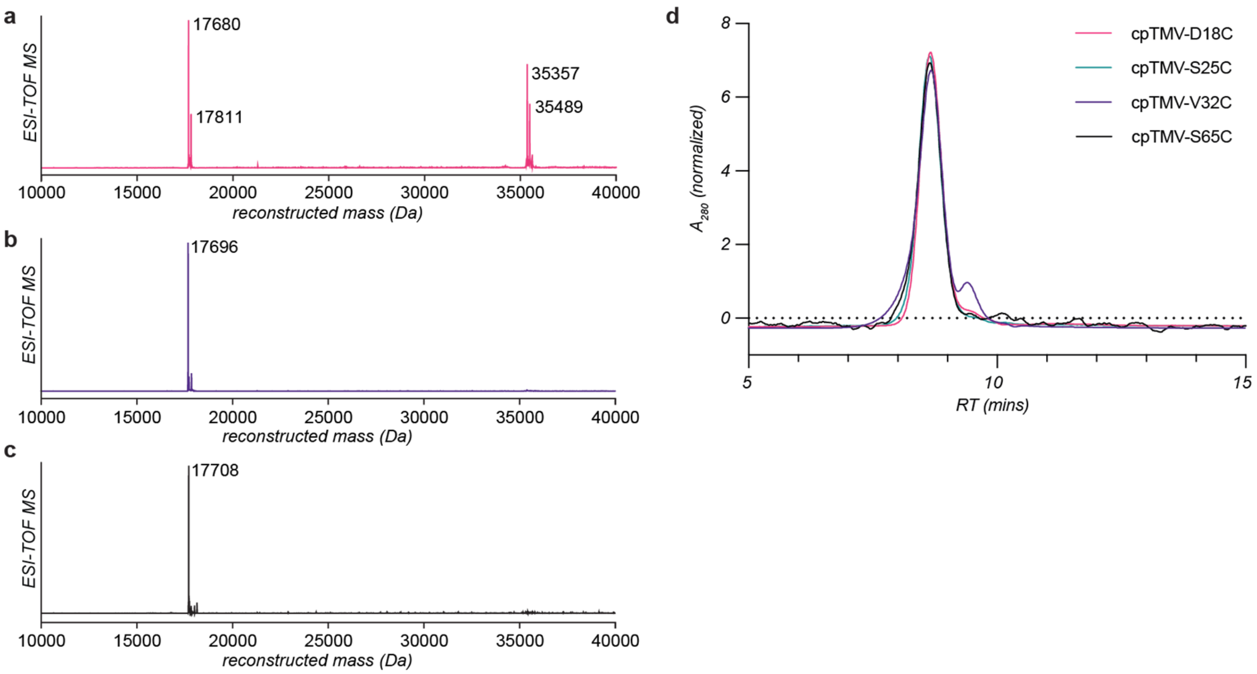

Figure S1: Characterization of new cpTMV mutants. (a) A mass spectrum of purified cpTMV-D18C shows the expected monomer MW of 17680 Da. The additional peak at 17811 Da is due to incomplete cleavage of the N-terminal methionine in vivo (expected MW: 17811 Da). This protein rapidly forms inter-monomer disulfide bonds upon exposure to air, resulting in dimerized cpTMV-D18C with complete N-terminal methionine cleavage (expected MW: 35358 Da) and dimers of monomers with and without the N-terminal methionine cleaved (expected MW: 35489 Da). (b) A mass spectrum of purified cpTMV-V32C shows the expected monomer MW of 17696 Da. (c) A mass spectrum of purified cpTMV-S65C shows the expected monomer MW of 17708 Da. (d) Overlaid size exclusion chromatography monitoring of the absorbance at 280 nm of cpTMV-D18C, cpTMV-V32C and cpTMV-S65C show that each mutant has a very similar retention time to the previously characterized cpTMV-S25C, indicating that each new mutant similarly forms a double disk structure consisting of 17 monomers per disk.

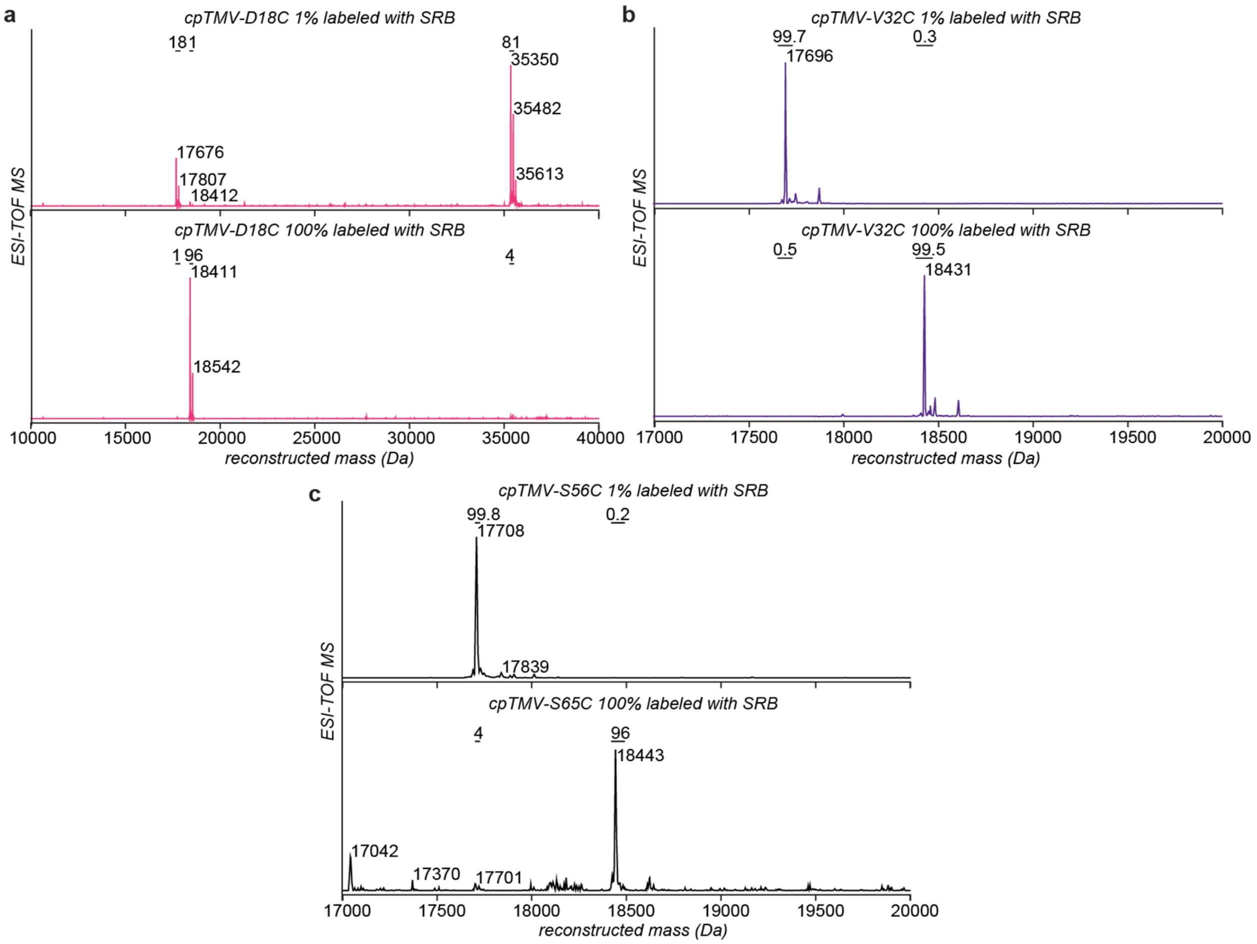

Figure S2: Labeling of cpTMV with maleimide derivatized sulforhodamine B. (a) Mass spectra show 1% and 100% labeled cpTMV-D18C (expected MW of single dye addition: 18414 Da). The additional peaks at 17811 and 18545 Da are due to incomplete cleavage of the N-terminal methionine in vivo (expected MW: 17811 and 18545 Da). A significant portion of dimerized protein containing a disulfide bond is also present in the 1% labeled sample. (b) Mass spectra show 1% and 100% labeled cpTMV-V32C (expected MW of single dye addition: 18430 Da). No appreciable disulfide bond formation and dimerization was observed for this mutant. (c) Mass spectra show 1% and 100% labeled cpTMV-S65C (expected MW of single dye addition: 18442 Da). No appreciable disulfide bond formation and dimerization was observed for this mutant.

2 Transient Absorption Anisotropy

This section builds on the information provided in the Methods to articulate additional intricacies about the setup and operation of the technique.

We measure the transient difference between pump-on and pump-off of the three signals from the BPD using three Stanford Research Systems SR830 lock-in amplifiers. The lock-in amplifiers are referenced to the chopping frequency of the pump, which was set to 1/8 of the laser repetition rate, i.e., 625 Hz. Thus we measure three quantities in the TAA experiment, each proportional to differences between beam intensities with the pump on ($I_{X,\mathrm{on}}$) and the pump off ($I_{X,\mathrm{off}}$) of the X polarization component (parallel, $\|$, and perpendicular, $\perp$): the two photodiode signals $\Delta P_+ \propto I_{\|,\mathrm{on}} - I_{\|,\mathrm{off}}$ and $\Delta P_- \propto I_{\perp,\mathrm{on}} - I_{\perp,\mathrm{off}}$, and the difference signal $\Delta D \propto (I_{\|,\mathrm{on}} - I_{\perp,\mathrm{on}}) - (I_{\|,\mathrm{off}} - I_{\perp,\mathrm{off}})$. The difference signal from the BPD is pre-amplified with a low-noise transimpedance amplifier, which allows much greater signal-to-noise ratio when the overall beam intensities are weak. Due to this amplification, however, the proportionality constant for ΔD differs from that of ΔP_+ and ΔP_- by some gain factor G, which we measure during an experiment by taking the ratio $\frac{\Delta D}{\Delta P_+ - \Delta P_-}$.

The expression for TA anisotropy is:

$$r = \frac{\Delta OD_{\|} - \Delta OD_{\perp}}{\Delta OD_{\|} + 2 \cdot \Delta OD_{\perp}}, \tag{S1}$$

where ΔOD_X is the change in the optical density, between pump-on and pump-off, of the X polarization component. For each polarization component, and assuming absorption is the only significant contribution to the optical density, this can be expressed in terms of probe intensities as $\Delta OD = OD_{\mathrm{on}} - OD_{\mathrm{off}} = -\log \frac{I_{\mathrm{on}}}{I_{0,\mathrm{on}}} + \log \frac{I_{\mathrm{off}}}{I_{0,\mathrm{off}}} = \log \frac{I_{\mathrm{off}}}{I_{\mathrm{on}}}$. For small changes in OD we can make the linear approximation $\log \frac{I_{\mathrm{off}}}{I_{\mathrm{on}}} \approx -\frac{I_{\mathrm{on}} - I_{\mathrm{off}}}{I_{\mathrm{off}}}$, omitting the factor of $\ln 10$ from the base 10 logarithm, as any such constant factor disappears in the expression for r. Substituting this into the expression above, we arrive at:

$$r \approx \frac{\frac{I_{\|,\mathrm{on}} - I_{\|,\mathrm{off}}}{I_{\|\mathrm{off}}} - \frac{I_{\perp,\mathrm{on}} - I_{\perp,\mathrm{off}}}{I_{\perp\mathrm{off}}}}{\frac{I_{\|,\mathrm{on}} - I_{\|,\mathrm{off}}}{I_{\|\mathrm{off}}} + 2 \cdot \frac{I_{\perp,\mathrm{on}} - I_{\perp,\mathrm{off}}}{I_{\perp\mathrm{off}}}} \tag{S2}$$

Last, because we carefully set the polarization of the probe such that the pump-off intensities of the two polarization components are equal, we can make the approximation that $I_{\|,\mathrm{off}} \approx I_{\perp,\mathrm{off}}$, and thus our expression becomes $r \approx \frac{(I_{\|,\mathrm{on}} - I_{\|,\mathrm{off}}) - (I_{\perp,\mathrm{on}} - I_{\perp,\mathrm{off}})}{(I_{\|,\mathrm{on}} - I_{\|,\mathrm{off}}) + 2(I_{\perp,\mathrm{on}} - I_{\perp,\mathrm{off}})}$. This can now be rewritten in terms of the three measured quantities and the gain factor defined previously:

$$r = \frac{1}{G} \cdot \frac{\Delta D}{\Delta P_+ + 2 \cdot \Delta P_-} \tag{S3}$$

Hence with a single pair of balanced photodetectors and three lock-in amplifiers we measure the transient absorption anisotropy.

3 Kinetic Monte Carlo Simulations

To simulate incoherent energy transfer in our statically disordered system, we represent an artificial light-harvesting complex as a ring of unit vectors each representing the orientation of the transition dipole moment (TDM) of a chromophore bound to the protein surface. These unit vectors are represented in a lab frame illustrated in **Fig. S4b**, where the z-axis is parallel to the polarization of the electric field of the pump. The orientation of each TDM in its site-local frame of reference (i.e., relative to the center of the ring, as shown in **Fig. S4b**) is selected via rejection sampling from a probability distribution $P(\theta, \phi)$ over polar angle θ and azimuthal angle ϕ. We perform simulations using two types of probability distributions: one obtained from molecular dynamics (MD) simulations and a Gaussian probability distribution, described below. Once the TDM orientations are selected, each site is assigned an energy drawn from a Gaussian distribution centered at the fundamental absorption energy $\tilde{\nu}_0$ and width σ_{ih}, the inhomogeneous broadening.

The TDM orientation probability distribution used to match the experimental data, which can be seen in the leftmost panel of **Fig. S4c**, is obtained from MD simulations of an individual chromophore covalently attached via a SS-cyclohexyl linker to residue 25 (as numbered in PDB 3KML) on three surface exposed α-helices of the biomimetic LHC.[2] The orientation of the TDM during the MD simulation is approximated by the orientation of the xanthene core[3] of the chromophore (specifically the vector from atom C12 to C11) in its site-local frame. To construct the site-local frame, illustrated for one site in **Fig. S4b**, the C atom of residue 25 and the N atom of residue 29 were used to define $\hat{x_{\text{site}}}$, and $\hat{z_{\text{site}}}$ as the vector normal to the plane that additionally contains the Cα atom of residue 25 (with all residue numbers as in PDB 3KML[4]). The distribution of θ and ϕ was constructed from the last 500 ns of the MD simulation. While experiments were performed on LHCs with the chromophore attached to a different, nearby residue, we do not expect this to significantly impact our findings since the experimental anisotropy curves are also well fit by sampling orientations from a Gaussian distribution with sufficient variance. Nevertheless, the distribution from the MD simulation captures the general influence of the linker, solvent, and protein environment.

To investigate the effects of TDM orientation disorder on excitation migration and anisotropy decay, we also run simulations using a Gaussian TDM orientation distribution. This orientation is defined with a central vector $\hat{\mu}_0$, chosen to be at the peak of the MD orientation distribution ($\theta_0 = 120°$, $\phi_0 = 105°$) and an angular width σ_α, which can be increased or decreased to respectively increase or decrease TDM orientational disorder. The probability $P_G(\theta, \phi)d\Omega$ for the TDM $\hat{\mu}$ defined by θ and ϕ to be drawn from this distribution, is defined as

$$P_G(\theta, \phi)d\Omega \propto e^{-\frac{\alpha^2}{2\sigma_\alpha^2}} \sin(\theta)d\theta d\phi, \tag{S4}$$

where α is the angle formed between $\hat{\mu}$ and $\hat{\mu}_0$, i.e. $\alpha = \arccos(\hat{\mu} \bullet \hat{\mu}_0)$, and the proportionality is set by normalization over the solid angle Ω. Two such orientation distributions for $\sigma_\alpha = 10°$ and $\sigma_\alpha = 90°$ are represented in false color in the right two panels of **Fig. S4c**. In the limit where $\sigma \to 0$ we obtain a Dirac delta function distribution where all TDMs on a ring point in the same direction in their site-local frame, while in the limit $\sigma \to \infty$ we obtain a uniform random TDM orientation distribution.

Once the rings are generated, they must be rotated randomly to simulate the random orientation of TMV rings in solution during the measurement. To do so, each ring is associated with a randomly-generated 3D rotation matrix. Because there is correlation between the different sites' orientations, care must be taken to do this correctly, as the selection of orientations must produce the same distribution one would expect from photoexcitation. In the dipole approximation, the probability of excitation at any site is proportional to the modulus square of the dot product of the transition dipole moment with the electric field, $|\hat{\mu} \cdot \vec{E}|^2$, or equivalently in this context μ_z^2 in the lab frame. For a dilutely excited population, where rings predominantly have one or zero excitations, the probability of a ring having one excitation is proportional to the average of all its member TDMs' alignments with the electric field:

$$P_{\text{ring}} \propto \frac{1}{N} \sum_{i=0}^{N-1} |\hat{\mu}_i \cdot \vec{E}|^2 \tag{S5}$$

where N is the number of sites on a ring and $\hat{\mu}_i$ is the TDM at the ith site. This is achieved by rejection sampling against the above probability criterion.

The rate $k_{i,j}$ between each site i and j on each ring is calculated using Förster theory:

$$k_{i,j} = k_F \cdot \frac{R_0^6}{r_{i,j}^6} \tag{S6}$$

where k_F is the fluorescence rate of SRB in the absence of FRET (1/1600 ps^{-1}), $r_{i,j}$ is the distance between the sites in nm, and R_0 is the FRET radius in nm, given by:

$$R_0^6 = \left(10^7 \frac{\text{nm}}{\text{cm}}\right)^6 \cdot \frac{9 \log 10 \cdot \Phi \cdot \kappa^2 \cdot J}{128\pi^5 \cdot N_A \cdot \eta^4} \tag{S7}$$

where $\Phi = 0.9$ is the fluorescence quantum yield of SRB, N_A is Avogadro's number, $\eta = 1.33$ is the refractive index of the solvent, taken to be that of water. κ^2 is the dipole orientation factor, which is computed from the two TDMs $\hat{\mu}_i$ and $\hat{\mu}_j$ and the unit vector $\hat{R}_{i,j}$ pointing from site i to site j:

$$\kappa^2 = \left(\hat{\mu}_i \bullet \hat{\mu}_j - 3 \cdot (\hat{\mu}_i \bullet \hat{R}_{i,j}) \cdot (\hat{\mu}_j \bullet \hat{R}_{i,j})\right)^2 \tag{S8}$$

Last, J is the overlap integral of the acceptor's molar absorptivity spectrum $\varepsilon_j(\tilde{\nu})$ and the donor's area-normalized fluorescence spectrum $\bar{F}(\tilde{\nu})$:

$$J = \int_0^\infty \bar{F}_i(\tilde{\nu}) \cdot \varepsilon_j(\tilde{\nu}) \cdot \tilde{\nu}^{-4} d\tilde{\nu} \tag{S9}$$

which, when evaluated over energy in wavenumbers (cm^{-1}) has units of $\text{cm}^6 \cdot \text{mol}^{-1}$. Our calculation of J builds on the method developed by Ahn *et al.*[5] to incorporate the vibronic progression and differing intrinsic linewidths.

For the simulation, the steady-state absorption and fluorescence spectra are fit by a sum of three Gaussian peaks, as shown in **Fig. S4d**, representing the fundamental ($\tilde{\nu}_0$) and two higher vibronic transitions. The fit for absorption is specified by the lowest absorption energy $\tilde{\nu}_0$, and for each peak (including the fundamental), an energy shift $\delta\tilde{\nu}_n$ relative to $\tilde{\nu}_0$, a width $\sigma_{\text{abs},n}$, and an absorption maximum $\varepsilon_{\text{abs},n}$ for each peak, where n indexes the peaks of the vibronic progression beginning at 0. The normalized fluorescence spectrum is fit by specifying a Stokes shift Δ_{SS} and a set of amplitudes $A_{\text{flu},m}$ for three peaks, otherwise using the same widths and energy shifts of the absorption peaks, in reversed energy order, thus assuming a perfectly mirror-imaged vibronic progression, which fits the spectrum reasonably well. A single inhomogeneous line width σ_{ih} is specified for all peaks, and the intrinsic linewidths σ_n for each peak are computed under a relation derived from the convolution of two Gaussians:

$$\sigma_n^2 = \sigma_{\text{abs},n}^2 - \sigma_{\text{ih}}^2 \tag{S10}$$

and likewise for intrinsic peak amplitudes:

$$\varepsilon_n = \varepsilon_{\text{abs},n} \cdot \frac{\sqrt{\sigma_n^2 + \sigma_{\text{ih}}^2}}{\sigma_n} \tag{S11}$$

and for intrinsic fluorescence amplitudes F_n. For the mth donor fluorescence peak and the nth acceptor absorption peak we define the respective peak energies $\tilde{\nu}_{m,i}$ and $\tilde{\nu}_{n,j}$ as follows:

$$\tilde{\nu}_{m,i} = \tilde{\nu}_{0,i} - \Delta_{SS} - \delta\tilde{\nu}_m \tag{S12}$$

$$\tilde{\nu}_{n,j} = \tilde{\nu}_{0,j} + \delta\tilde{\nu}_n. \tag{S13}$$

Thus for each site an intrinsic absorption and fluorescence spectrum is constructed, using these intrinsic parameters as illustrated in **Fig. S4e** for a donor and acceptor pair of chromophores.

Then, the total overlap integral, shown as the pink shaded region in **Fig. S4e**, between sites i and j is given by a sum of overlap integrals over each pair of peaks:

$$\sum_{n=0}^{2} \sum_{m=0}^{2} \int_0^\infty F_{i,m} \cdot \exp\left(-\frac{(\tilde{\nu} - \tilde{\nu}_{m,i})^2}{2\sigma_m^2}\right) \cdot \varepsilon_{j,n} \cdot \exp\left(-\frac{(\tilde{\nu} - \tilde{\nu}_{n,j})^2}{2\sigma_n^2}\right) \cdot \tilde{\nu}^{-4} d\tilde{\nu}. \tag{S14}$$

The product of two Gaussians itself is Gaussian and thus the integrand of Eqn. S14 can be rewritten as a the product of a single Gaussian and $\tilde{\nu}^{-4}$:

$$\sum_{n=0}^{2}\sum_{m=0}^{2} F_{i,m} \cdot \varepsilon_{j,n} \cdot \exp\left(-\frac{(\tilde{\nu}_{n,j}-\tilde{\nu}_{m,i})^2}{2(\sigma_m^2+\sigma_n^2)}\right) \cdot \int_0^\infty \exp\left(\frac{1}{2}\cdot\frac{\sigma_m^2+\sigma_n^2}{\sigma_m^2\cdot\sigma_n^2}\cdot\left(\tilde{\nu}-\frac{\sigma_n^2\tilde{\nu}_{m,i}+\sigma_m^2\tilde{\nu}_{n,j}}{\sigma_m^2+\sigma_n^2}\right)^2\right)\cdot\tilde{\nu}^{-4}d\tilde{\nu} \tag{S15}$$

Eqn. S15 does not have an analytical solution; thus we make an approximation that the $\tilde{\nu}^{-4}$ term is constant over the width of the Gaussian, which is valid when the width is much smaller than the energy at the peak. So, we treat the $\tilde{\nu}^{-4}$ term as constant when evaluated at the mean of the Gaussian, allowing analytical integration of the expression:

$$\sum_{n=0}^{2}\sum_{m=0}^{2} F_{i,m} \cdot \varepsilon_{j,n} \cdot \exp\left(-\frac{(\tilde{\nu}_{n,j}-\tilde{\nu}_{m,i})^2}{2(\sigma_m^2+\sigma_n^2)}\right)\cdot\left(\frac{\sigma_m^2+\sigma_n^2}{\sigma_n^2\tilde{\nu}_{m,i}+\sigma_m^2\tilde{\nu}_{n,j}}\right)^4 \cdot\sigma_m\cdot\sigma_n\sqrt{\frac{2\pi}{\sigma_m^2+\sigma_n^2}} \tag{S16}$$

With this expression we efficiently compute J for all pairs of sites on each ring.

An excitation is placed on each ring by randomly selecting a site with probability proportional its TDM's alignment with the $\hat{z}$-aligned electric field, i.e., $|\hat{\mu}\bullet\hat{z}|^2$. We then propagate the system in a kinetic Monte Carlo scheme using an implementation of the Gillespie algorithm.[6,7,8] For each ring we keep 1D arrays of each time at which the excitation hopped, the index of the site hopped to, and a tally of the excitation displacement in units of number of sites, with counterclockwise hops counted positively and *vice versa*. Each trajectory ends after 5 ns have elapsed.

Once all trajectories have ended, we compute the anisotropy of the population at any time t as:

$$r(t) = \frac{3\left\langle\mu_{n,z}^{*2}\right\rangle - 1}{2} \tag{S17}$$

where $\mu_{n,z}^{*}$ is the z-component of the TDM occupied by the excitation on the nth ring at time t, and $\langle\rangle$ denotes averaging over the population. Similarly, the mean squared displacement (MSD) is defined using the displacement:

$$MSD(t) = \left\langle d_n(t)^2\right\rangle \tag{S18}$$

where $d_n(t)$ is the displacement of the excitation on the nth ring at time t. Lastly we compute the time-dependent fluorescence spectrum of the excited population as:

$$F(\tilde{\nu},t) = \langle IRF(t) * F_n(\tilde{\nu},t)\rangle \tag{S19}$$

where $IRF(t)$ is the Gaussian instrument response function, and F_n is the fluorescence spectrum of the excited chromophore at time t.

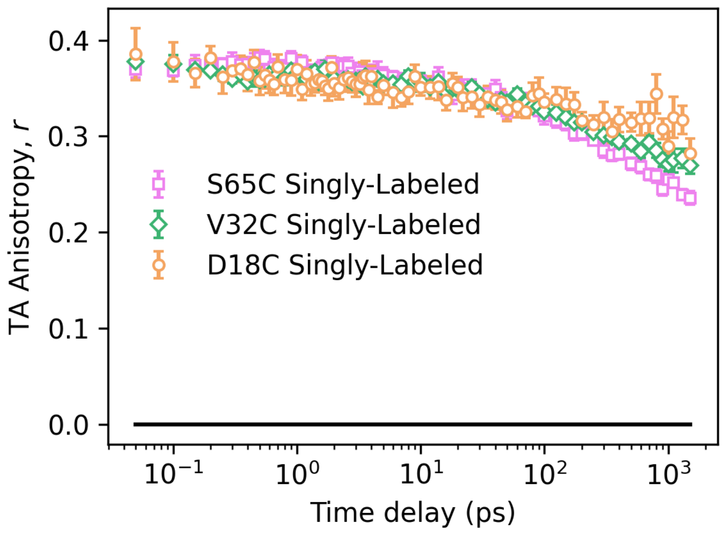

Figure S3: TAA data of singly-labeled control samples at different sites on the protein surface, with anisotropy decay least prevalent on D18C complexes indicating highest site rigidity.

	A_1	τ_1 (ps)	A_2	τ_2 (ps)	A_3	τ_3 (ps)
Fully-labeled LHC	0.33	1.5	0.31	98	0.35	870
Singly-labeled control	0.15	4.4	—	—	0.84	1800
Free SRB control	0.15	6.6	—	—	0.87	1600

Table S1: Multiexponential reconvolution fits for magic-angle TA kinetics as shown in Fig. S6. Fit equation is of the form $\Delta OD(t) = IRF(t) * \left(\sum_n A_n \cdot e^{-(t-t_0)/\tau_n}\right)$, where n indexes exponential components, t_0 is the pump-probe overlap time (not shown, typically $|t_0|<50$ fs), and $IRF(t)$ is the instrument response function, assumed to be Gaussian.

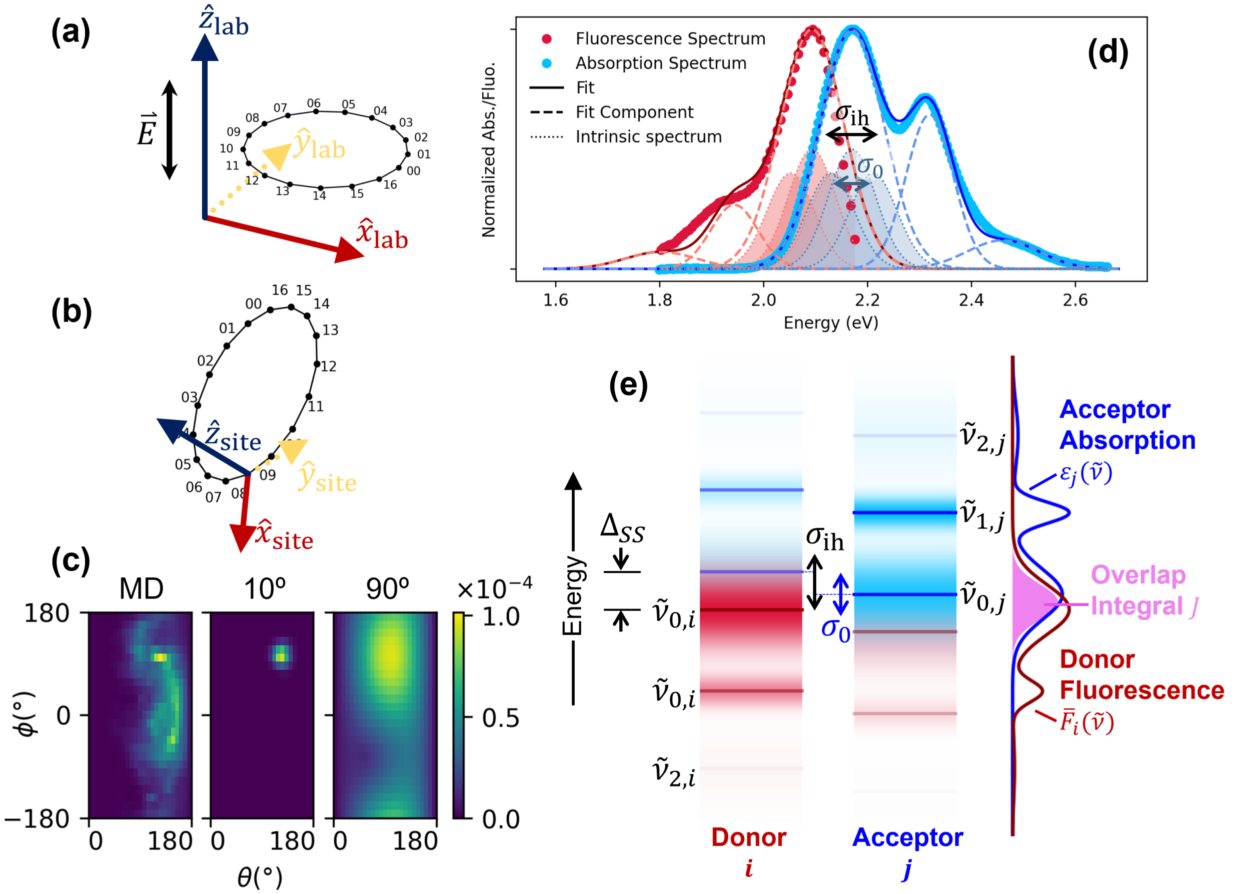

Figure S4: The orientational and energetic parameters passed to the kinetic Monte Carlo simulation. (a) The lab frame of the simulation, where the electric field $\vec{E}$ is parallel to the z-axis $\hat{z}_{\mathrm{lab}}$. (b) The local site frame where the x-axis $\hat{x}_{\mathrm{site}}$ points outward from the center of the ring. In this coordinate frame the orientations are randomly generated from an orientation probability distribution, as shown in false color in (c), obtained from molecular dynamics simulations (left panel) or using a Gaussian distribution (right two panels, for two widths σ_α). (d) The Gaussian fits to the absorption and emission spectra of the LHC, with the inhomogeneous linewidth σ_{ih} and intrinsic linewidth σ_0 represented; from this fit we derive the parameters shown in (e) which are used in the computation of the spectral overlap integral J (see Eqn. S16).

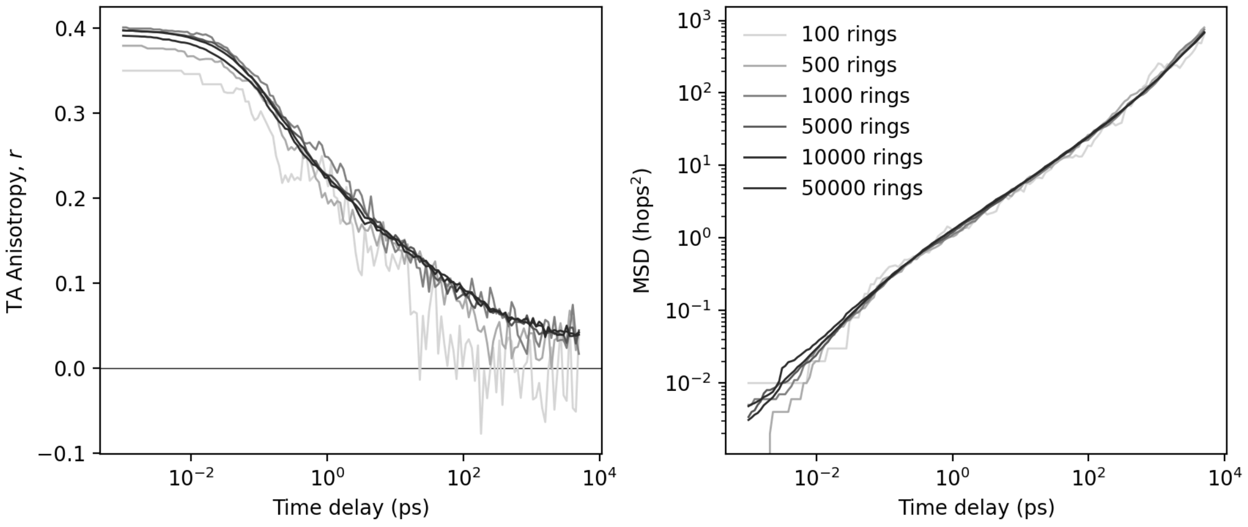

Figure S5: Convergence of the computed observables, anisotropy (top) and MSD (bottom), to a reproducible result occurs above a population size of 5000 for a 17-membered ring with disorder included.

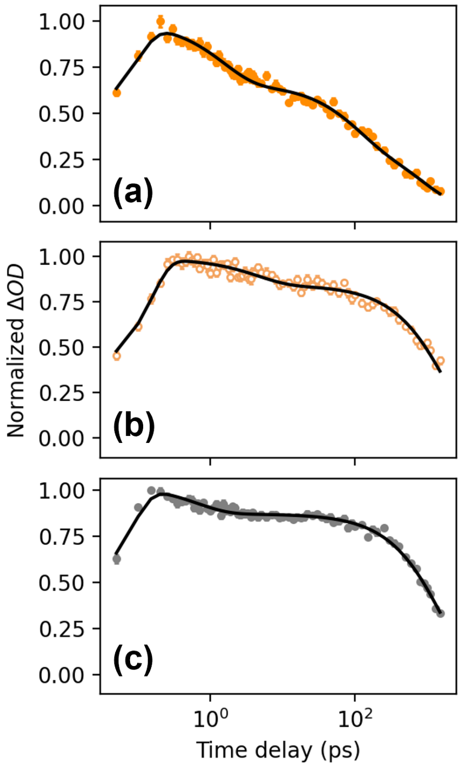

Figure S6: Magic-angle TA kinetics of (a) fully-labeled LHC, (b) singly-labeled control, and (c) free SRB control at 580 nm, with multiexponential reconvolution fits overlaid; see fit parameters in **Table S1**.

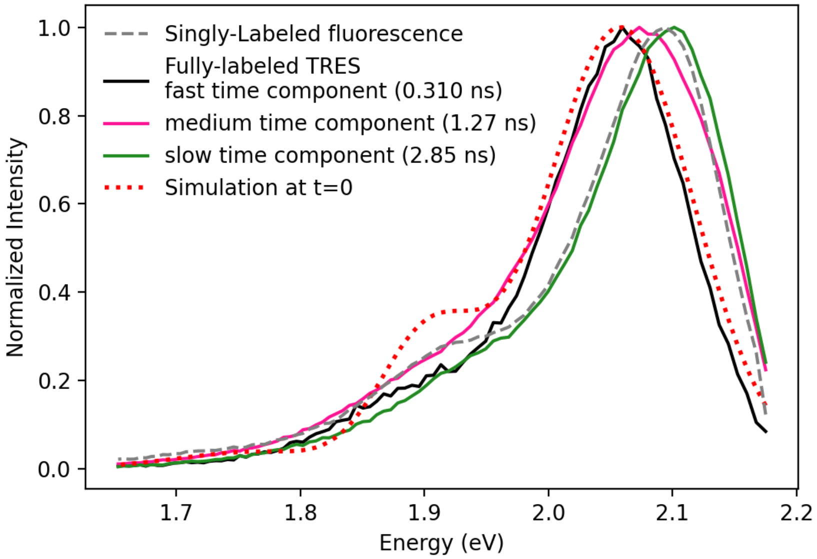

Figure S7: Components of triexponential fit to TRES of fully-labeled LHC (solid curves) compared to the early time spectrum of simulated population convolved with a 150 ps Gaussian IRF (dotted red), and to the late-time spectrum of singly-labeled control complexes (dashed gray).

References

(1) Dedeo, M. T.; Duderstadt, K. E.; Berger, J. M.; Francis, M. B. *Nano Letters* **2010**, *10*, Publisher: American Chemical Society, 181–186.

(2) Delor, M.; Dai, J.; Roberts, T. D.; Rogers, J. R.; Hamed, S. M.; Neaton, J. B.; Geissler, P. L.; Francis, M. B.; Ginsberg, N. S. *Journal of the American Chemical Society* **2018**, *140*, 6278–6287.

(3) Penzkofer, A.; Wiedmann, J. *Optics Communications* **1980**, *35*, 81–86.

(4) Miller, R. A.; Presley, A. D.; Francis, M. B. *Journal of the American Chemical Society* **2007**, *129*, 3104–3109.

(5) Ahn, T.-S.; Wright, N.; Bardeen, C. J. *Chemical Physics Letters* **2007**, *446*, 43–48.

(6) Gillespie, D. T. *Journal of Computational Physics* **1976**, *22*, 403–434.

(7) Gillespie, D. T. *The Journal of Physical Chemistry* **1977**, *81*, Publisher: American Chemical Society, 2340–2361.

(8) Gillespie, D. T. *Annual Review of Physical Chemistry* **2007**, *58*, 35–55.
